# Integrin β1 regulates marginal zone B cell differentiation and PI3K signaling

**DOI:** 10.1084/jem.20220342

**Published:** 2022-11-09

**Authors:** Virginia Andreani, Senthilkumar Ramamoorthy, Reinhard Fässler, Rudolf Grosschedl

**Affiliations:** 1 Max Planck Institute of Immunobiology and Epigenetics, Freiburg, Germany; 2 Institute for Immunodeficiency, Center for Chronic Immunodeficiency, Medical Center, University of Freiburg, Freiburg, Germany; 3 Institute of Medical Bioinformatics and Systems Medicine, Medical Center, University of Freiburg, Freiburg, Germany; 4 Division of Pediatric Hematology and Oncology, Department of Pediatrics and Adolescent Medicine, Medical Center, University of Freiburg, Freiburg, Germany; 5 Max Planck Institute of Biochemistry, Martinsried, Germany

## Abstract

Marginal zone (MZ) B cells represent innate-like B cells that mediate a fast immune response. The adhesion of MZ B cells to the marginal sinus of the spleen is governed by integrins. Here, we address the question of whether β1-integrin has additional functions by analyzing *Itgb1*^fl/fl^*CD21*^Cre^ mice in which the β1-integrin gene is deleted in mature B cells. We find that integrin β1–deficient mice have a defect in the differentiation of MZ B cells and plasma cells. We show that integrin β1–deficient transitional B cells, representing the precursors of MZ B cells, have enhanced B cell receptor (BCR) signaling, altered PI3K and Ras/ERK pathways, and an enhanced interaction of integrin-linked kinase (ILK) with the adaptor protein Grb2. Moreover, the MZ B cell defect of integrin β1–deficient mice could, at least in part, be restored by a pharmacological inhibition of the PI3K pathway. Thus, β1-integrin has an unexpected function in the differentiation and function of MZ B cells.

## Teaser

Integrins mediate the localization of innate-like B cells to the marginal zone of the spleen. By analyzing mice with β1-integrin deficiency in mature B cells, Andreani et al. show that β1-integrin is also required for normal differentiation and function of marginal zone B cells.

## Introduction

Integrins are heterodimeric cell-surface receptors composed of α and β subunits. They mediate cell adhesion to extracellular matrix proteins, including collagen and laminin, and cell–cell adhesion by binding receptors such as vascular cell adhesion molecule-1 (VCAM-1) and intracellular adhesion molecule-1. Integrins can sense the biochemical and biophysical properties of the substrate and transduce this information into signaling pathways that in turn regulate adhesion strength, polarity, migration, survival, and proliferation of cells (Hynes, 2002; Luo et al., 2007; Moser et al., 2009). Mammals produce 18 α and 8 β subunits that can form 24 integrin heterodimers with specific ligand binding and signaling properties. The β1-integrin subunit can associate with 12 α subunits, and hence forms the largest integrin subfamily (Hynes, 2002). The association of β1 and α4 produces α4β1, also known as VLA-4 (very late antigen-4), which is particularly abundant on lymphocytes and binds VCAM-1 and fibronectin. The α4β1 integrin regulates different processes in leukocytes including cell adhesion and cell trafficking to different organs and inflamed tissues (Kinashi, 2005). The B cell localization in the splenic marginal zone (MZ) and the peripheral lymphoid tissue compartmentalization are orchestrated by the extracellular matrix (Song et al., 2013) and by integrin interactions with VCAM-1, which is abundantly expressed in the red pulp of the spleen (Lu and Cyster, 2002; Ulyanova et al., 2005).

Peripheral B lymphocytes consist of multiple cell populations that differ in their phenotype, functional properties, and anatomic locations (Allman and Pillai, 2008; Martin and Kearney, 2000; Cerutti et al., 2013). Follicular B (Fo B) cells, representing the majority of peripheral B cells, are localized in lymph nodes and follicles of the spleen. They engage predominantly in a slow but highly specific T cell–dependent (TD) immune response. In contrast, MZ B cells are localized in the MZ of the spleen, where they rapidly respond to T cell–independent (TI) antigens of bloodborne pathogens, including bacterial LPS. The specific splenic location of murine MZ B cells is a consequence of the abundant expression of α4β1 and αLβ2 integrins which, together with the integrin α6β1 and the S1p1 receptor, allow these cells to adhere to this anatomical structure (Lu and Cyster, 2002; Cinamon et al., 2004; Song et al., 2013).

In the bone marrow (BM) of adult mice, B cell differentiation generates immature B cells that have successfully completed Ig heavy- and light-chain gene rearrangements and display an IgM-class B cell receptor (BCR) on the cell surface. Cells that pass the negative selection checkpoint for self-reactive BCRs transit to the spleen where they further differentiate via transitional (T1–T3) B cell stages to generate either mature Fo B cells or MZ B cells (Loder et al., 1999; Chung et al. 2003; Hardy et al., 2007; Lindsley et al., 2007). The commitment toward one of these mature peripheral B cell types depends on differences in the threshold of BCR signaling and the combination with other signaling pathways. Fo B cell differentiation is favored by strong BCR signals and the activation of the B cell growth factor (BAFF)–induced prosurvival pathway. On the other hand, commitment to MZ B cell differentiation is governed by weak IgM-BCR signals and the activation of the Notch2 signaling pathway via the interaction of transitional B cells with the Dll1 ligand on stromal cells of the spleen (Pillai and Cariappa, 2009). In addition to the well-documented function of integrins for the adhesion and localization of MZ B cells to the MZ of the spleen, integrin signaling has been proposed to contribute to the BCR- and Notch2-driven differentiation of MZ B cells (Pillai and Cariappa, 2009). However, no experimental evidence for a role of integrins in MZ B cell differentiation has yet been addressed.

The role of the β1-class integrins in lymphocytes has been studied in different mouse models (Hirsch et al., 1996; Brakebusch et al., 2002; Nandi et al., 2004). We and others reported the relevance of β1-class integrins for the migration of plasma cells (PCs) to the BM (van Spriel, et al., 2012; Andreani et al., 2018; Saveliev et al., 2021). To better understand β1-class integrin functions in mature B cell biology, we generated mice lacking β1-integrin expression on CD21^+^ cells. We report that β1-integrin has also a role in MZ B cell differentiation and find that this process is associated with BCR signaling and with the PI3K and Ras pathways. We show that the pharmacological inhibition of the PI3K pathway restores, at least in part, the MZ B cell phenotype, and that during MZ B cell differentiation, many of the genes deregulated in β1-integrin–deficient CD21^+^ cells belong to the family of Ras-GTPase–related proteins. In addition, we find that integrin-related proteins interact with Ras-GTPase–related proteins in β1-integrin–deficient transitional B cells. Our findings uncover a novel and important role of β1-integrin in MZ B cell differentiation and function.

## Results

### MZ B cell numbers in *β1*^*fl/fl*^CD21-cre mice are reduced despite splenic retention

**Figure 1.**
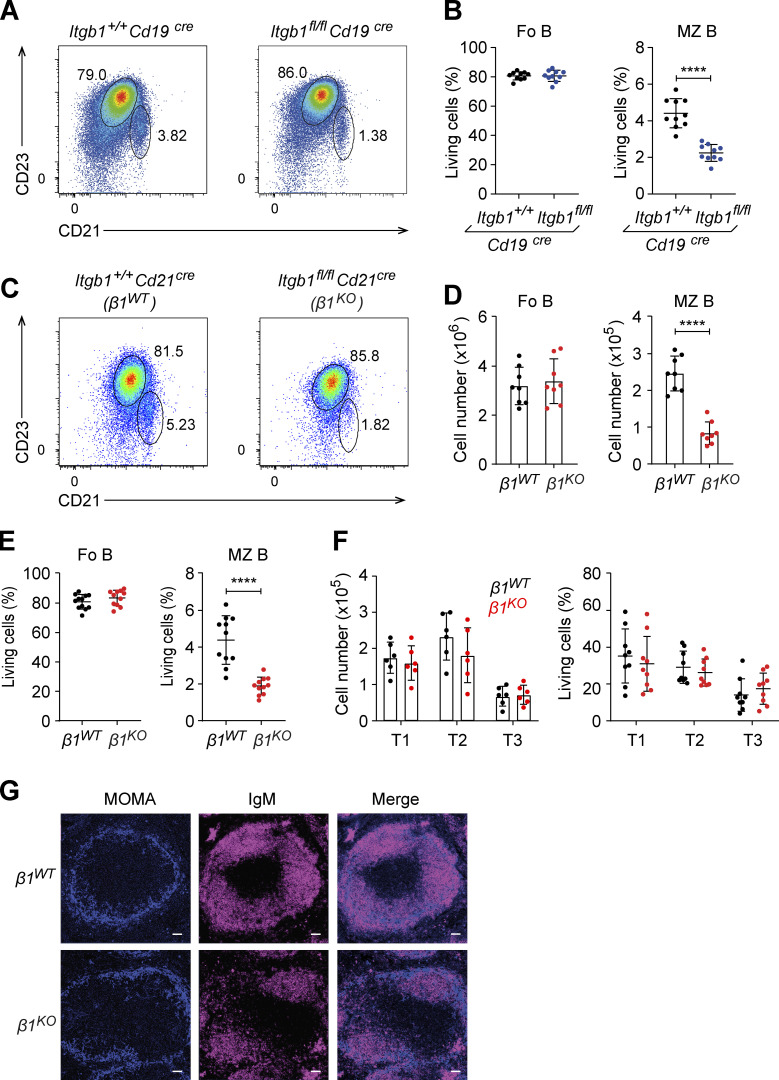
**MZ B cell frequencies are reduced in *β1***^***KO***^ **mice. (A)** Flow cytometry to identify Fo (CD23^hi^CD21^low^) and MZ B cells (CD23^low^CD21^hi^) in spleen from *Itgb1*^*+/+*^*Cd19*^*cre*^ and *Itgb1*^*fl/fl*^*Cd19*^*cre*^ mice. Numbers represent cell frequencies. **(B)** Mean (±SD) frequencies of Fo and MZ B cells in spleen from *Itgb1*^*+/+*^*Cd19*^*cre*^ and *Itgb1*^*fl/fl*^*Cd19*^*cre*^ mice, as gated in A. **(C)** Flow cytometry to identify Fo (CD23^hi^CD21^low^) and MZ B cells (CD23^low^CD21^hi^) in spleen from *Itgb1*^*+/+*^*Cd21*^*cre*^ (*β1*^*WT*^) and *Itgb1*^*fl/fl*^*Cd21*^*cre*^ (*β1*^*KO*^) mice. **(** **and** **E)** Numbers represent cell frequencies. Mean (±SD) frequencies (D) and absolute numbers (E) of Fo and MZ B cells in spleen from *β1*^*WT*^ and *β1*^*KO*^ mice, as gated in C. **(F)** Mean (±SD) absolute numbers and frequencies of transitional B cells (T1-T3) from *β1*^*WT*^ and *β1*^*KO*^ mice. **(A–F)** *n* = 5–9 mice. Each circle in the graphs represents data from one mouse. Data are representative of five different experiments. Mean and SD are indicated by horizontal lines in the data points; significance is calculated by unpaired Student’s *t* test (****P < 0.0001). **(G)** Immunofluorescence staining for MOMA to define the MZ (blue) and IgM (red) for B cells in the spleens of *β1*^*WT*^ and *β1*^*KO*^ mice. *n* = 3 mice. Scale bars, 100 µm.

To evaluate the role of β1-integrin in the differentiation and function of MZ B cells, we used two mouse models for the Cre-mediated deletion of the β1-integrin gene *Itgb1*. First, we crossed mice carrying floxed alleles of *Itgb1* with CD19-cre mice that express the Cre recombinase specifically in the B cell lineage (Rickert et al., 1997). By a flow cytometric analysis of splenic B cells, we observed significantly reduced frequencies of CD19^+^CD93^−^CD23^−^CD21^hi^ MZ B cells in *Itgb1*^fl/fl^*Cd19*^Cre^ mice relative to *Itgb1*^+/+^*Cd19*^Cre^ mice, whereas the frequencies of CD19^+^CD93^−^CD23^hi^CD21^int^ Fo B cells were not altered (Fig. 1, A and B). Secondly, we crossed the mice carrying floxed alleles of *Itgb1* with CD21-cre mice that mediated deletion in transitional and mature B cells (Kraus et al., 2004). In the spleen of *Itgb1*^fl/fl^*Cd21*^Cre^ mice, referred to as *β1*^*KO*^ mice, we detected a similar reduction in the numbers and frequencies of MZ B cells relative to *Itgb1*^+/+^*Cd21*^Cre^ mice, referred to as *β1*^*WT*^ mice (Fig. 1, C–E).

**Figure S1.**
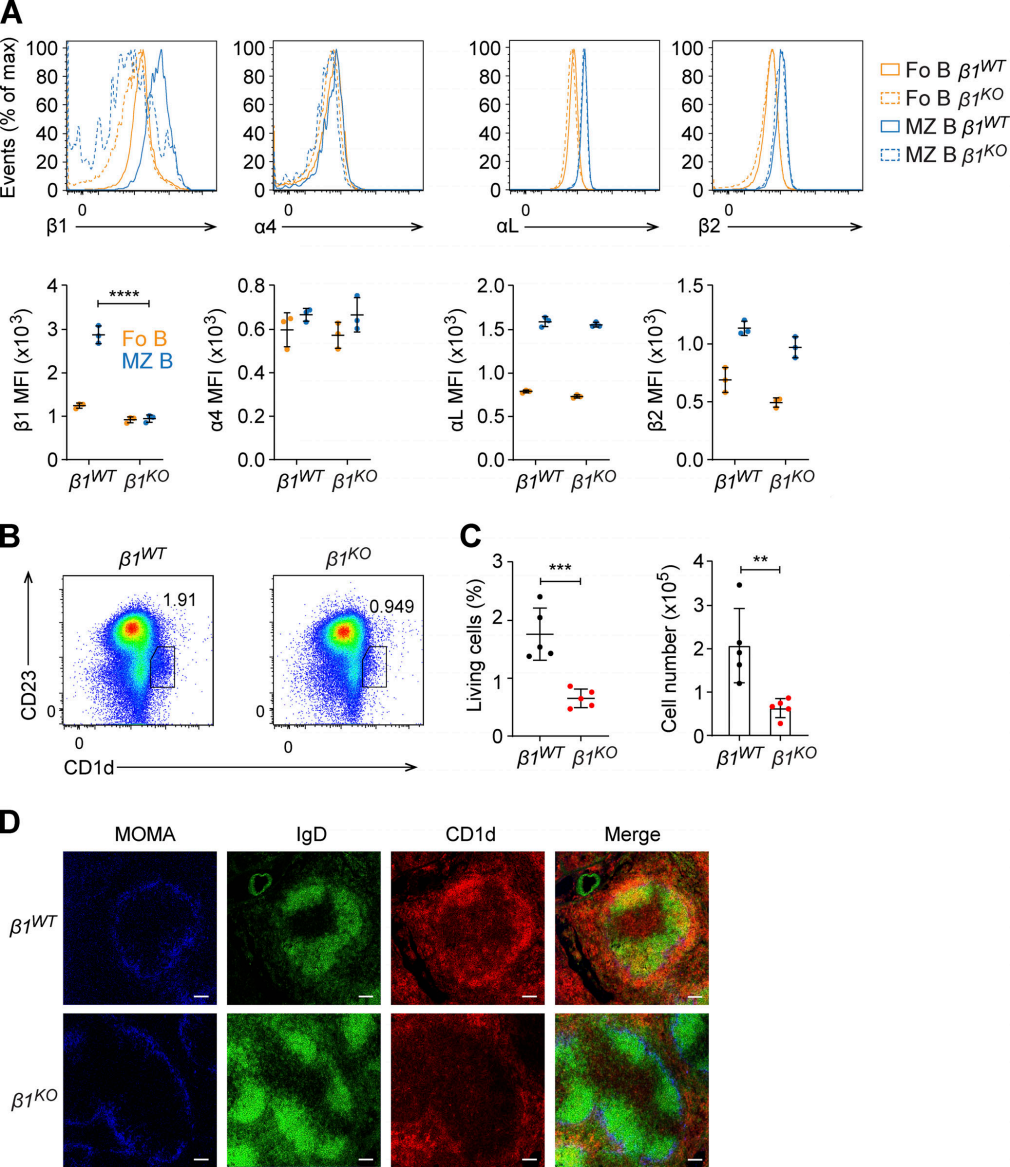
**Reduced β1-integrin expression and reduced MZ B cell numbers in *β1***^***KO***^ **mice.** Related to Fig. 1. **(A)** Histograms analysis (upper panels) and MFI quantification (lower panels) of β1-, α4-, αL-, and β2-integrins in Fo (orange) and MZ (blue) B cells from *β1*^*WT*^ and *β1*^*KO*^ mice. **(B)** Flow cytometry to identify Fo (CD23^hi^CD1d^low^) and MZ B cells (CD23^low^CD1d^hi^) in spleen from *β1*^*WT*^ and *β1*^*KO*^ mice. **(C)** Mean (±SD) frequencies and absolute numbers of Fo and MZ B cells in spleen from *β1*^*WT*^ and *β1*^*KO*^ mice, as gated in B. **(A–C)** *n* = 3–5 mice. Each circle in the graphs represents data from one animal. Data are representative of four independent experiments. Mean and SD are indicated by horizontal lines in the data points; significance is calculated by unpaired Student’s *t* test (**P < 0.01, ***P < 0.001, ****P < 0.0001). **(D)** Immunofluorescence staining for MOMA to define the MZ (blue), IgD (green) for B cells and CD1d (red) for MZ B cells, in the spleens of *β1*^*WT*^ and *β1*^*KO*^ mice. *n* = 3 mice. Scale bars, 100 µm.

Flow cytometric analysis of β1 integrin expression in splenic B cells showed markedly reduced levels of β1 integrin on the surface of *β1*^*KO*^ MZ B cells relative to the corresponding cells of *β1*^*WT*^ mice (Fig. S1 A). Moreover, the levels of β1-integrin on Fo B cells, which are much lower than those on MZ B cells (Pillai and Cariappa, 2009), were further reduced in *β1*^*KO*^ mice relative to *β1*^*WT*^ mice (Fig. S1 A). In MZ B cells, β1-integrin forms a heterodimer with the α4-integrin (VLA-4), which provides these cells together with the αLβ2 (LFA-1) heterodimer their sessile, nonrecirculatory state (Lu and Cyster, 2002). Therefore, we also evaluated the surface expression of α4-, αL-, and β2-integrins on Fo B and MZ B cells of *β1*^*KO*^ and *β1*^*WT*^ mice by flow cytometry. In both cell types, we observed similar surface expression of α4-, αL-, and β2-integrins in *β1*^*KO*^ and *β1*^*WT*^ MZ B cells (Fig. S1 A), raising the possibility that α4-integrin may be paired with another β-integrin in *β1*^*KO*^ cells.

Among the CD19^+^ splenic B cells, the expression of CD93 defines the transitional B cells that can be further subdivided into CD93^+^IgM^hi^CD23^−^ T1 cells, CD93^+^IgM^hi^CD23^hi^ T2 cells, and CD93^+^IgM^low^CD23^hi^ T3 cells. The absolute numbers and frequencies of all three transitional B cell populations were unchanged in *β1*^*KO*^ mice, indicating that the deletion of β1-integrin affects specifically the MZ B cell population (Fig. 1 F). The reduced MZ B cell population in *β1*^*KO*^ mice was confirmed by the additional flow cytometric analysis of CD1d^+^ cells, another surface marker of MZ B cells (Fig. S1, B and C).

Examination of the histological architecture of the spleens of *β1*^*WT*^ and *β1*^*KO*^ mice indicated that the marginal zone, visualized by the staining for metallophilic macrophages (MOMA), is markedly reduced in the *β1*^*KO*^ mice (Fig. 1 G). The spleens of *β1*^*KO*^ mice also had reduced staining of IgM^hi^ B cells. Additional immunostaining of spleen sections, aimed at identifying CD1d^+^ MZ B cells and IgD^+^ B cells, indicated a specific reduction of CD1d^+^ MZ B cells in *β1*^*KO*^ mice (Fig. S1 D). Thus, the deletion of the β1-integrin gene in CD21^+^ cells leads to a reduction of the MZ B cell population in the spleen.

**Figure S2.**
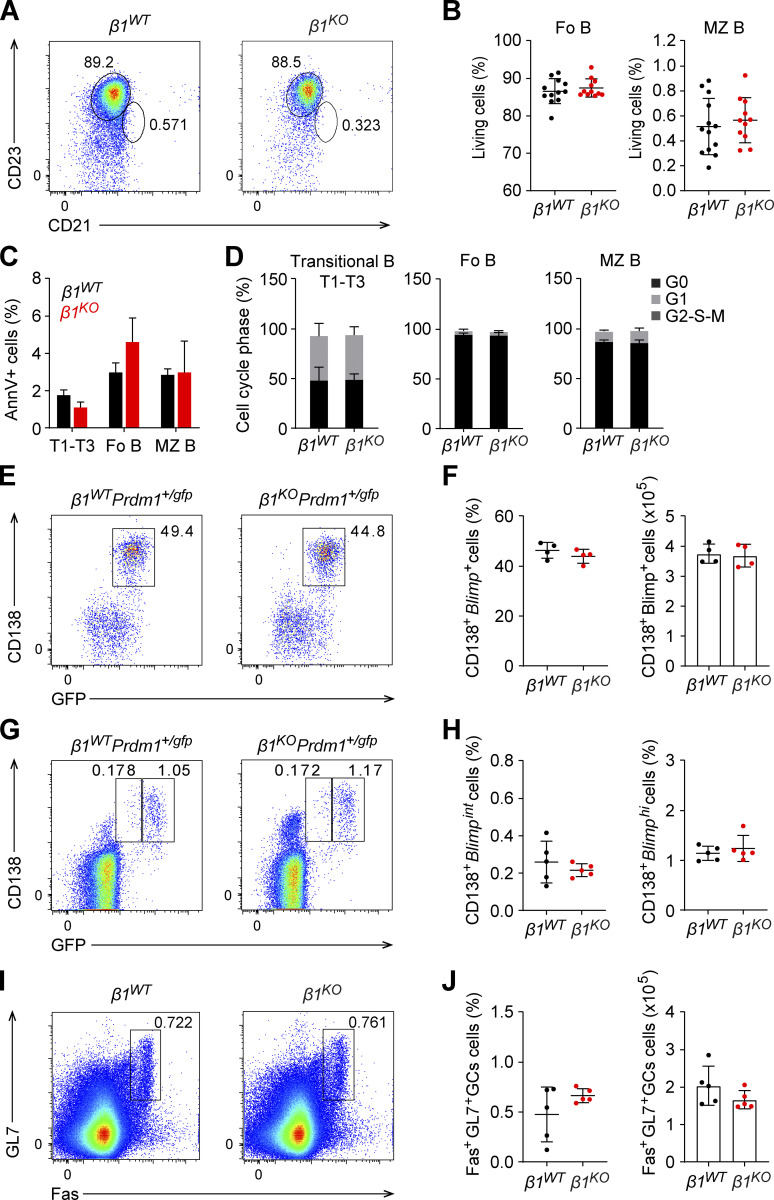
***β1***^***KO***^ **mice have normal PC differentiation in TD immune responses.** Related to Fig. 2. **(A)** Flow cytometry analysis of *β1*^*WT*^ and *β1*^*KO*^ peripheral blood cells showing the Fo (CD23^hi^CD21^low^) and MZ (CD23^low^CD21^hi^) B cell populations. **(B)** Mean (±SD) frequencies of Fo and MZ B cells in peripheral blood of *β1*^*WT*^ and *β1*^*KO*^ mice, as gated in A. **(C)** Mean (±SD) frequencies of transitional (T1–T3), Fo, and MZ B Annexin V^+^ cells in spleens of *β1*^*WT*^ and *β1*^*KO*^ mice. **(D)** Mean (±SD) frequencies within each cell cycle phase of transitional (T1–T3), Fo, and MZ B from spleens of *β1*^*WT*^ and *β1*^*KO*^ mice. **(A–D)** *n* = 3–13 mice. Each circle in the graphs represents data from one animal. **(E)** Flow cytometry to identify CD138^+^Blimp-GFP^+^ cells among B220^+^ *β1*^*WT*^Prdm1^+/gfp^ and *β1*^*KO*^Prdm1^+/gfp^ splenocytes that were stimulated with CD40L, IL4, and IL5 in vitro for 5 d. Numbers represent cell frequencies. **(F)** Mean (±SD) frequencies and numbers of CD138^+^Blimp-GFP^+^ cells, as gated in E. **(G)** Flow cytometry to detect CD138^+^Blimp-GFP^+^ cells in the BM of *β1*^*WT*^Prdm1^+/gfp^ and *β1*^*KO*^ Prdm1^+/gfp^ mice 7 d p.i. with NP-KLH. Numbers represent cell frequencies. **(H)** Mean (±SD) frequencies of *β1*^*WT*^ and *β1*^*KO*^ CD138^+^Blimp-GFP^int^ and CD138^+^Blimp-GFP^hi^ cells in BM, as gated in G. **(I and J)** Flow cytometry to determine the frequencies of GC B cells in *β1*^*WT*^ and *β1*^*KO*^ mice 7 d p.i. with NP-KLH. Representative dot plots (I) and mean (±SD) frequencies and numbers (J) of GL7^+^ Fas^+^ GC B cells in spleens of NP-KLH immunized mice. *n* = 3–5 mice. Each circle in the graphs represents data from one animal. Mean and SD are indicated by horizontal lines in the data points. Data are representative of three independent experiments.

Integrins contribute to the retention of MZ B cells in the MZ of the spleen, as determined by the marked increase of MZ B cells in peripheral blood after the combined inhibition of αLβ2 (LFA) and α4β1 integrins (Lu and Cyster, 2002). Therefore, we examined whether or not the absence of β1-integrin in MZ B cells is sufficient for their release from the spleen. Similarly, low frequencies of circulating MZ B cells were detected in the peripheral blood of *β1*^*KO*^ and *β1*^*WT*^ mice, suggesting that the lack of β1-integrin is not sufficient to mobilize splenic MZ B cells (Fig. S2, A and B). Moreover, we detected no changes in the survival or proliferation of MZ B cells, transitional, and Fo B cells in the spleen of *β1*^*KO*^ mice relative to *β1*^*WT*^ mice (Fig. S2, C and D). Taken together, these data suggest that abundant β1-integrin expression is required for the accumulation of MZ B cells in the spleen, in addition to the well-documented role of integrins for their retention in the spleen.

### TI humoral immune responses and PC differentiation are impaired in *β1*^*KO*^ mice

**Figure 2.**
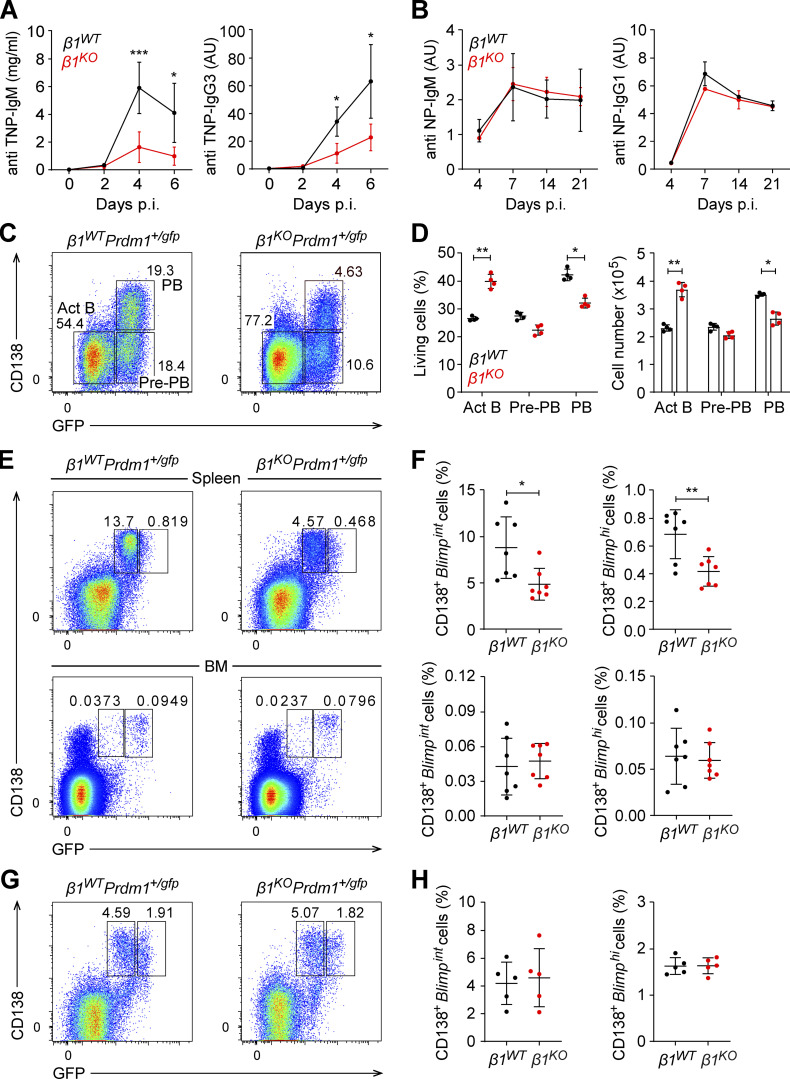
**β1-integrin regulates TI PC differentiation. (A)** IgM (left panel) and IgG3 (right panel) serum levels in *β1*^*WT*^ and *β1*^*KO*^ mice at different time points p.i. with TNP-LPS. **(B)** IgM (left panel) and IgG1 (right panel) serum levels in *β1*^*WT*^ and *β1*^*KO*^ mice at different time points p.i. with NP-KLH. **(A and B)** *n* = 5 mice. Data are from three independent experiments. **(C)** Flow cytometry to identify CD138^−^Blimp-GFP^−^ Act B cells, CD138^−^Blimp-GFP^+^ pre-PBs, and CD138^+^Blimp-GFP^+^ PBs 4 d after LPS stimulation of *β1*^*WT*^Prdm1^+/gfp^ and *β1*^*KO*^Prdm1^+/gfp^ B220^+^ splenocytes. Numbers represent cell frequencies. **(D)** Mean (±SD) frequencies and absolute numbers of the populations gated in C*.* **(E)** Flow cytometry to detect CD138^+^Blimp-GFP^+^ cells in the spleen (upper) and BM (lower) of *β1*^*WT*^Prdm1^+/gfp^ and *β1*^*KO*^Prdm1^+/gfp^ mice 3 d p.i. with TNP-LPS. Numbers represent cell frequencies. **(F)** Mean (±SD) frequencies of *β1*^*WT*^ and *β1*^*KO*^ CD138^+^Blimp-GFP^int^ and CD138^+^Blimp-GFP^hi^ cells in the spleen and BM, as gated in E. **(G)** Flow cytometry to identify CD138^+^Blimp-GFP^+^ cells in spleen cells from *β1*^*WT*^Prdm1^+/gfp^ and *β1*^*KO*^Prdm1^+/gfp^ mice 7 d p.i. with NP-KLH. Numbers represent cell frequencies. **(H)** Mean (±SD) frequencies of CD138^+^Blimp-GFP^int^ and CD138^+^Blimp-GFP^hi^ cells in spleens from *β1*^*WT*^Prdm1^+/gfp^ and *β1*^*KO*^Prdm1^+/gfp^ mice, as gated in G. **(C–H)** *n* = 4–7 mice. Each circle in the graphs represents data from one animal. Data are representative of three independent experiments. Mean and SD are indicated by horizontal lines in the data points; significance is calculated by one-way ANOVA test (*P < 0.05, **P < 0.01, ***P < 0.001).

MZ B cells respond rapidly to TI antigens, such as bacterial LPS, but they can also engage in a slower TD immune response that is primarily mediated by Fo B cells (Cerutti et al., 2013). These processes commence with the differentiation of short-lived, cycling, antibody-secreting plasmablasts (PBs) that further differentiate into quiescent, long-lived PCs to provide long-term immunity (Nutt et al., 2015). To evaluate a potential role of β1-integrin in the humoral immune response, we immunized *β1*^*WT*^ and *β1*^*KO*^ mice with the TI antigen trinitrophenyl-LPS (TNP-LPS) and subsequently analyzed TNP-specific IgM and IgG3 serum levels. *β1*^*WT*^ mice mounted a robust anti-TNP immune response, whereas the levels of TNP-specific IgM and IgG3 antibodies were markedly reduced in *β1*^*KO*^ mice (Fig. 2 A). However, *β1*^*KO*^ mice responded as efficiently as WT mice to the immunization with the TD antigen 4-hydroxy-3-nitrophenyl-acetyl-keyhole limpet hemocyanin (NP-KLH; Fig. 2 B). These findings indicate that the lack of β1-integrin in CD21^+^ cells results in an impaired humoral immune response specifically against TI antigens.

To examine the effects of the β1-integrin deletion on PC differentiation, we crossed *β1*^*KO*^ mice with *Prdm1*^*+/gfp*^ mice in which GFP reports on the expression of the transcription factor Blimp1 (Kallies et al., 2004). Blimp1 is expressed at intermediate levels in PBs and high levels in PCs, allowing for the identification and separation of short-lived, cycling Blimp1^int^ PBs from long-lived, quiescent Blimp1^hi^ PCs in vivo (Kallies et al., 2004). Moreover, the combined analysis of Blimp1-GFP and CD138 enables the analysis of the differentiation of activated B (Act B) cells (CD138^−^ Blimp1-GFP^−^), pre-PBs (CD138^−^ Blimp1-GFP^+^), and PBs (CD138^+^Blimp1-GFP^+^) in vitro (Kallies et al., 2004). First, we examined the differentiation of LPS-treated B220^+^ splenocytes in vitro by using flow cytometric analysis to detect Act B cells, pre-PBs, and PBs. The frequencies and absolute numbers of PBs were significantly decreased in *β1*^*KO*^*Prdm1*^+/gfp^ relative to *β1*^*WT*^*Prdm1*^+/gfp^ mice, whereas the frequencies and numbers of Act B cells were increased in the *β1* mutant mice (Fig. 2, C and D). In contrast, in vitro stimulation of B220^+^ splenic cells with CD40L, IL4, and IL5, which mimics TD PC differentiation, yielded similar frequencies of CD138^+^ Blimp1-GFP^+^ PBs in cultures from *β1*^*KO*^*Prdm1*^+/gfp^ and *β1*^*WT*^*Prdm1*^+/gfp^ mice (Fig. S2, E and F). Thus, the β1-integrin deficiency results in an impaired in vitro differentiation of Act B cells and pre-PBs to PBs, specifically in response to TI stimulation.

As the in vitro differentiation fails to generate terminally differentiated PCs (Nutt et al., 2015; Shi et al., 2015), we also examined differentiation in vivo by immunizing *β1*^*WT*^*Prdm1*^*+/gfp*^ and *β1*^*KO*^*Prdm1*^*+/gfp*^ mice with TNP-LPS. Flow cytometric analysis of B220^+^ splenocytes at 3 d post immunization (p.i.) indicated that the frequencies of both CD138^+^Blimp-GFP^int^ PBs and CD138^+^Blimp1-GFP^hi^ PCs were reduced in *β1*^*KO*^*Prdm1*^*+/gfp*^ mice compared with *β1*^*WT*^*Prdm1*^*+/gfp*^ mice (Fig. 2, E and F). However, the frequencies of both populations were similar in the BM of *β1*^*WT*^*Prdm1*^*+/gfp*^ and *β1*^*KO*^*Prdm1*^*+/gfp*^ mice (Fig. 2, E and F), suggesting that the β1-integrin deficiency impairs PB and PC differentiation but does not affect the accumulation of these cells in the BM.

To confirm the specific role of β1-integrin in TI PC generation, we also immunized *β1*^*WT*^*Prdm1*^*+/gfp*^ and *β1*^*KO*^*Prdm1*^*+/gfp*^ mice with NP-KLH, which elicits a TD immune response. At 7 d p.i., the frequencies of CD138^+^ Blimp-GFP^int^ PB and CD138^+^ Blimp1-GFP^hi^ PC were similar in both spleen (Fig. 2, G and H) and BM (Fig. S2, G and H) of *β1*^*WT*^*Prdm1*^*+/gfp*^ and *β1*^*KO*^*Prdm1*^*+/gfp*^ mice. Moreover, germinal center (GC) B cells were detected at similar frequencies in *β1*^*WT*^ and *β1*^*KO*^ mice after immunization with NP-KLH (Fig. S2, I and J). Taken together, these results indicate that β1-integrin deficiency in CD21^+^ B cells leads to impaired PC differentiation specifically upon TI antigen exposure.

### β1-integrin–deficient transitional and MZ B cells have altered transcriptional profiles

**Figure 3.**
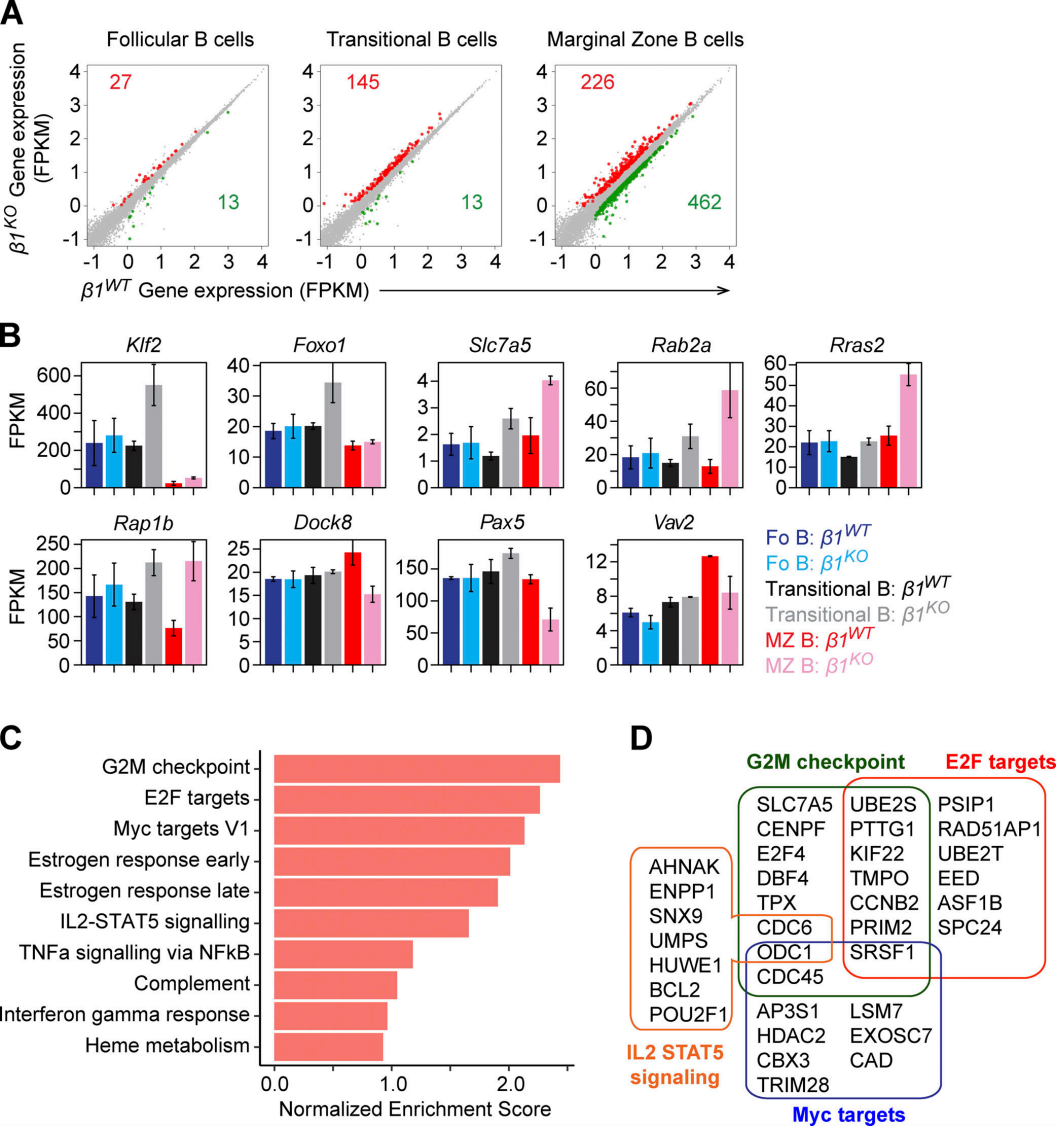
**β1-integrin affects the transcriptional program of MZ B cell differentiation. (A)** Scatter plot of gene-expression levels in *β1*^*WT*^ (x axis) and *β1*^*KO*^ (y axis) Fo B (left), transitional B (center), and MZ B (right) cells. The unaltered (gray), up- (red), and downregulated (green) genes are highlighted. **(B)** Expression levels (fragments per kilobase of transcript per million mapped reads [FPKM]) of differentially expressed key genes in *β1*^*WT*^ and *β1*^*KO*^ Fo B, transitional B, and MZ B cells. Error bars indicate SD; *n* = 3. **(C)** The top 10 enriched hallmark gene sets (y axis) in the differentially expressed genes of *β1*^*KO*^ MZ B cells relative to *β1*^*WT*^ MZ B cells. Gene sets are ordered by normalized enrichment score (x axis). **(D)** List and overlap of enriched genes (from C) in the hallmark gene sets G2M checkpoint, E2F targets, Myc targets, and IL2-STAT5 pathway. RNA-seq was performed once.

To gain insight into the molecular basis of the reduced frequencies and antigen-driven differentiation of MZ B cells in β1-integrin–deficient mice, we performed a genome-wide transcriptome analysis of ex vivo–sorted Fo B, transitional B, and MZ B cells. RNA sequencing (RNA-seq) analysis of these cell populations in *β1*^*KO*^ and *β1*^*WT*^ mice identified 145 upregulated and 13 downregulated genes in *β1*^*KO*^ transitional B cells relative to *β1*^*WT*^ transitional B cells (Fig. 3 A). In *β1*^*KO*^ MZ B cells, 226 genes were upregulated and 462 genes were downregulated as compared with *β1*^*WT*^ MZ B cells. In contrast, only 40 genes were up- or downregulated in *β1*^*KO*^ Fo B cells (Fig. 3 A). Interestingly, the transcription factor genes *Klf2* and *Foxo1*, whose genetic deletion induces an expansion of the MZ B cell compartment (Chen et al., 2010; Hart et al., 2011; Winkelmann et al., 2011), were significantly upregulated in both *β1*^*KO*^ transitional and MZ B cells (Fig. 3 B). The comparative analysis of genes that were upregulated in both *β1*^*KO*^ transitional and MZ B cells showed an overlapping set of 87 genes that included *Slc7a5*, encoding a large neutral amino acids transporter associated with ERK activation in MZ B cells (Cantor et al., 2009; Sintes et al., 2017), as well as *Rab2a*, *Rras2*, and *Rap1b*, encoding members of the Ras family of small guanosine triphosphatases (GTPases; Fig. 3 B and Data S1). Of note, *Rap1b* deletion generates a loss of MZ B cells (Chen et al., 2008; Su et al., 2015), and various GTPases have been associated with the regulation of MZ B cells and PCs (Guinamard et al., 2000; Chen et al., 2016; Ortega-Molina et al., 2021). β1-integrin–deficient MZ B cells showed an additional downregulation of genes, including *Dock8*, *Pax5*, and *Vav2*, which was not observed in *β1*^*KO*^ transitional B cells (Fig. 3 B), suggesting that the defects are enhanced in differentiated MZ B cells. In comparison with *β1*^*WT*^ MZ B cells, *β1*^*KO*^ MZ B cells also showed an increase in PI3K/mTORC1-coordinated gene sets associated with cell proliferation (G2M checkpoint, E2F targets, and Myc targets) and cell activation (IL2-STAT5 signaling and TNFα signaling via NF-κB; Fig. 3, C and D). Notably, these and other deregulated genes showed similar expression in *β1*^*KO*^ Fo B cells and *β1*^*WT*^ Fo B cells (Fig. 3 B). Thus, this analysis indicates transitional B and MZ B cell–specific changes in the expression of genes connected to the Ras/ERK and PI3K/mTORC1 signaling pathways in the absence of β1-integrin.

### β1-integrin–deficient transitional B cells show increased BCR signaling

**Figure 4.**
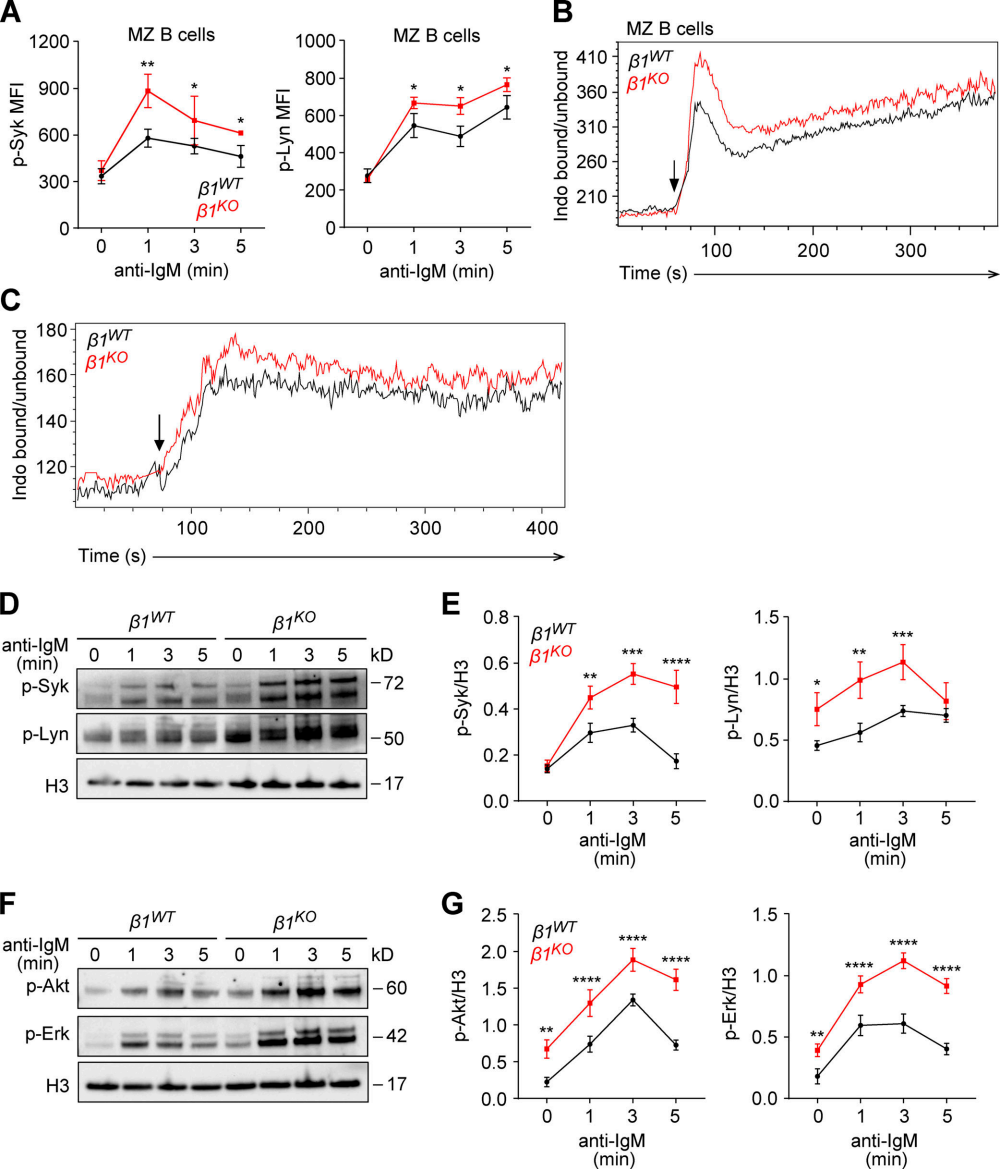
**β1-integrin regulates BCR signaling in transitional B cells. (A)** MZ B cells from *β1*^*WT*^ and *β1*^*KO*^ mice were stimulated for different time points with anti-mouse IgM F(ab′)2 antibody and the phosphorylation of Syk (left) and Lyn (right) was evaluated by flow cytometry. Mean (±SD) of mean fluorescence intensity (MFI) is plotted. **(B and C)** Ca^2+^ influx was measured in *β1*^*WT*^ and *β1*^*KO*^ MZ (B) and transitional (C) B cells. Cells were stained with Indo-1 and the ratio of Ca^2+^-bound Indo-1 to Ca^2+^-unbound Indo-1 was measured by flow cytometry. Stimulation was induced after 60 s of the measurement with anti-IgM (5 µg/ml; arrow). Data are representative of three independent experiments. **(D)** *β1*^*WT*^ and *β1*^*KO*^ transitional B cells were stimulated for different time points with anti-mouse IgM F(ab′)2 antibody and the phosphorylation of Syk and Lyn was evaluated by Western blot. **(E)** A quantification of the phospho-Syk (left) and phospho-Lyn (right)/loading control ratio after normalization is shown for independently performed experiments as in D. The mean ± SD is plotted. **(F)** *β1*^*WT*^ and *β1*^*KO*^ transitional B cells were stimulated for different time points with anti-mouse IgM F(ab′)2 antibody and the phosphorylation of Akt and Erk was evaluated by Western blot. **(G)** A quantification of the phospho-Akt (left) and phospho-Erk (right)/loading control ratio after normalization is shown for independently performed experiments as in F. The mean ± SD is plotted. **(A–G)** *n* = 3–5 mice. Data are representative of three independent experiments. Significance was calculated by one-way ANOVA test (*P < 0.05, **P < 0.01, ***P < 0.001, ****P < 0.0001). Source data are available for this figure: SourceData F4.

**Figure S3.**
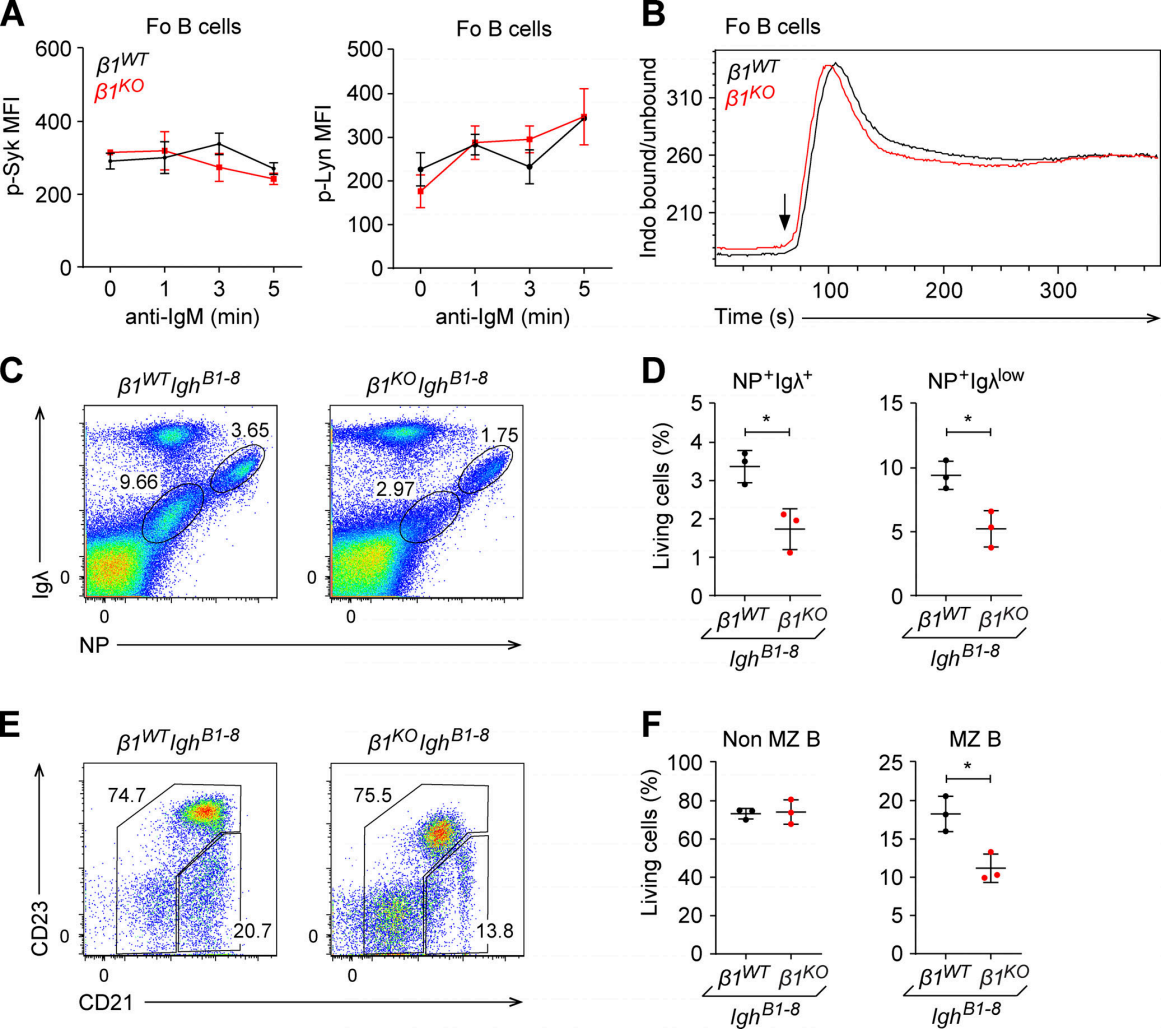
***β1***^***KO***^ **MZ B cells have altered self-antigen recognition.** Related to Fig. 4. **(A)** Fo B cells from *β1*^*WT*^ and *β1*^*KO*^ mice were stimulated for different time points with anti-mouse IgM F(ab′)2 antibody and the phosphorylation of Syk (left) and Lyn (right) was evaluated by flow cytometry. Mean (±SD) of MFI is plotted. **(B)** Ca^2+^ influx was measured in *β1*^*WT*^ and *β1*^*KO*^ Fo B cells. Cells were stained with Indo-1 and the ratio of Ca^2+^-bound Indo-1 to Ca^2+^-unbound Indo-1 was measured by flow cytometry. Stimulation was induced after 60 s of the measurement with anti-IgM (5 µg/ml; arrow). **(C and D)** Flow cytometry (C) and mean (±SD) frequencies (D) of NP^+^ Igλ^+^ and NP^+^ Igλ^low^ cells in the spleen of *β1*^*WT*^*Igh*^*B1-8*^ and *β1*^*KO*^*Igh*^*B1-8*^ mice. **(E and F)** Flow cytometry (E) and mean (±SD) frequencies (F) of non-MZ (CD23^hi^CD21^low^) and MZ B (CD23^low^CD21^hi^) cells gated in the NP^+^ Igλ^low^ population of *β1*^*WT*^*Igh*^*B1-8*^ and *β1*^*KO*^*Igh*^*B1-8*^ mice. **(A–F)** *n* = 3–5 mice. Each circle in the graphs represents data from one animal. Data are representative of three different experiments. Mean and SD are indicated by horizontal lines in the data points; significance is calculated by unpaired Student’s *t* test (*P < 0.05).

The upregulation of Ras family genes in *β1*^*KO*^ MZ B cells and in vitro–differentiated transitional B cells, together with the known association of Ras proteins with BCR and GTPase signaling (Oh-hora et al., 2003; Aiba et al., 2004), lead us to investigate the BCR signaling response. First, we analyzed the phosphorylation of the tyrosine kinases Syk and Lyn, which are recruited to the BCR and activated after antigen binding or crosslinking with anti-IgM (Kurosaki, 1999; Rolli et al., 2002). To this end, we stimulated MZ B cells with anti-mouse F(ab′)2 fragments and performed a flow cytometric analysis to detect the phosphorylated forms of Syk and Lyn. After 1 min of anti-IgM-mediated BCR stimulation, phosphorylation of both tyrosine kinases was increased in *β1*^*KO*^ MZ B cells, relative to *β1*^*WT*^ MZ B cells (Fig. 4 A). Second, we analyzed anti-IgM induced Ca^2+^ signaling and found augmented Ca^2+^ mobilization in *β1*^*KO*^ MZ B cells and transitional B cells relative to their WT counterparts (Fig. 4, B and C). We also analyzed the phosphorylation of Syk and Lyn in *β1*^*KO*^ and *β1*^*WT*^ transitional B cells by immunoblot analysis (Fig. 4 D). This analysis and the quantification of the data from multiple experiments indicated that the phosphorylation of Syk and Lyn was increased in *β1*^*KO*^ relative to *β1*^*WT*^ transitional B cells, whereby the phosphorylation of Lyn was already increased in *β1*^*KO*^ cells prior to BCR stimulation (Fig. 4, D and E). Third, we measured the BCR-induced activation of the phosphoinositide 3-kinase (PI3K) pathway, as determined by the phosphorylation of the Akt kinase (Otero et al., 2001) and the Ras pathway, by analyzing the phosphorylation of Erk. The PI3K pathway acts downstream of the BCR and regulates early B cell differentiation and the establishment and maintenance of late B cells (Yasuda et al., 2008; Srinivasan et al., 2009; Rowland et al., 2010; Werner et al., 2010). Likewise, the Ras/Erk pathway has been linked to BCR signaling (Niiro and Clark, 2002; Oh-hora et al., 2003; Coughlin et al., 2005). Immunoblot analysis of Akt and Erk phosphorylation after BCR ligation showed significantly higher levels of phosphorylation in *β1*^*KO*^ transitional B cells as compared to *β1*^*WT*^ transitional B cells (Fig. 4, F and G). In contrast to the enhanced BCR and PI3K signaling in *β1*^*KO*^ transitional and MZ B cells, *β1*^*KO*^ Fo B cells showed similar anti-IgM-induced phosphorylation of Syk and Lyn as compared with *β1*^*WT*^ Fo B cells (Fig. S3 A). Likewise, the BCR-induced Ca^2+^ mobilization in *β1*^*KO*^ and *β1*^*WT*^ Fo B cells was similar (Fig. S3 B). Taken together, these data suggest that β1-integrin regulates BCR, PI3K, and Ras signaling specifically in transitional and MZ B cells.

It has been proposed that self-ligand-mediated positive selection can occur at the immature stage of B cell development, and in particular during the generation of MZ B cells (Martin and Kearney, 2000). To evaluate whether β1-integrin influences self-antigen recognition, we crossed *β1*^*WT*^ and *β1*^*KO*^ mice with mice carrying the rearranged Igh B1-8 heavy-chain allele (*Igh*^B1-8^ mice), which in association with the Ig λ light chain generates a BCR specific for NP (Sonoda et al., 1997). These mice have a subset of NP hapten-recognizing B cells with Igλ on the surface (NP^+^ Igλ^+^) and an NP-recognizing B cell population with an Igλ^low^ surface phenotype (NP^+^ Igλ^low^) that shows a stronger BCR activation and increased self-reactivity relative to NP^+^ Igλ^+^ B cells (Noviski et al., 2019). By flow cytometric analysis of splenic B cells for NP-recognizing cells, we observed a marked decrease of both Igλ^+^ NP^+^ and Igλ^low^ NP^+^ cell populations in *β1*^*KO*^ *Igh*^B1-8^ mice relative to both cell populations in *β1*^*WT*^*Igh*^B1-8^ mice (Fig. S3, C and D). Furthermore, flow cytometric analysis of MZ B cells indicated a decrease of MZ B cells in *β1*^*KO*^*Igh*^B1-8^ mice as compared with *β1*^*WT*^*Igh*^B1-8^ mice (Fig. S3, E and F). Together, these data suggest that the β1-integrin–mediated regulation of BCR signaling may also affect the self-antigen recognition in MZ B cells.

### Cell-intrinsic function of β1-integrin in MZ B cell differentiation

**Figure 5.**
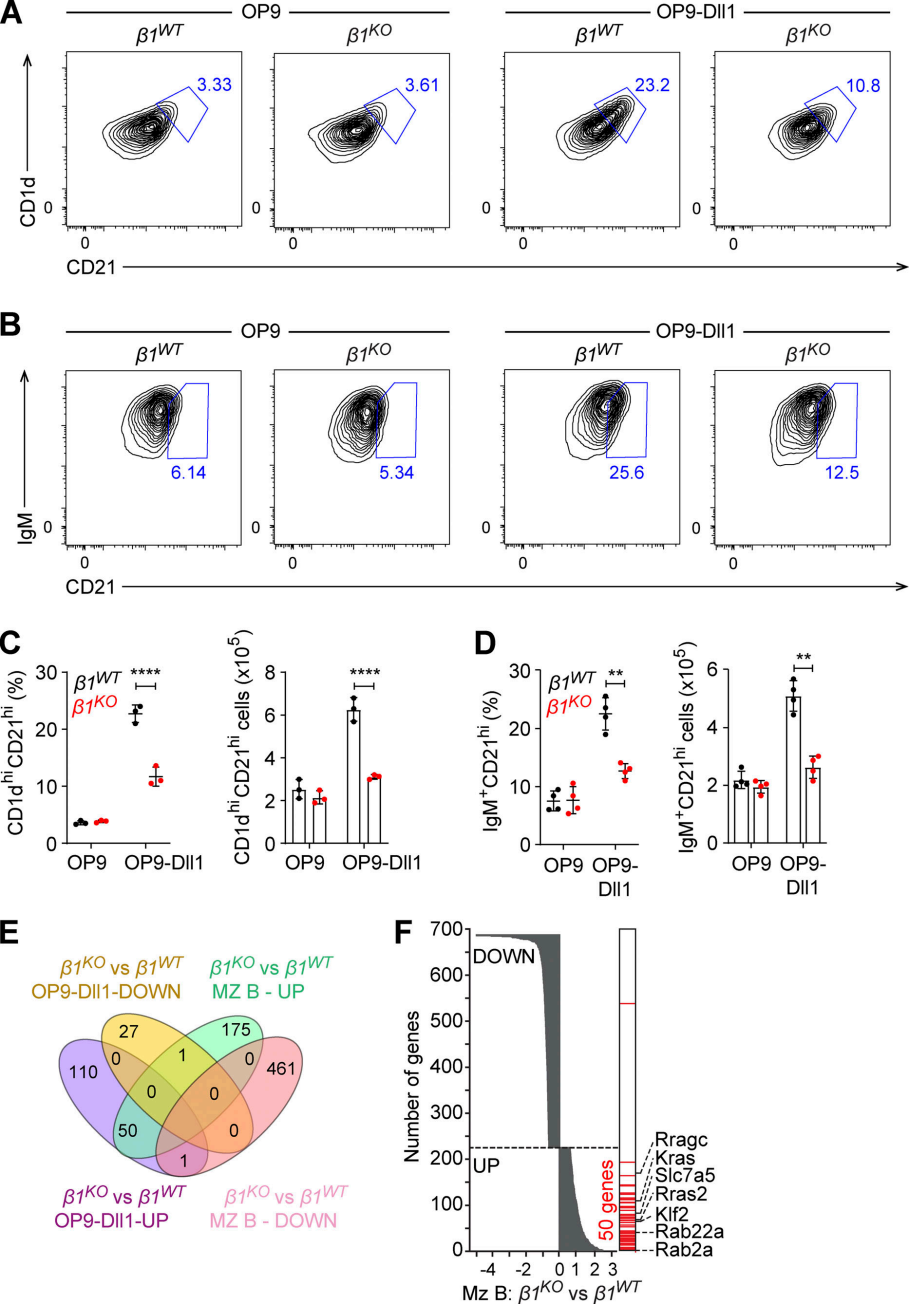
**β1-integrin regulates MZ B cell differentiation. (A and B)** Flow cytometry to identify the induction of CD1d and CD21 (A) and IgM and CD21 (B) expression on transitional B cells from *β1*^*WT*^ and *β1*^*KO*^ mice, cultured on OP9 or OP9-Dll1 cells for 3 d. **(C and D)** Mean (±SD) frequencies and absolute numbers of the increase in CD1d^hi^ CD21^hi^ (C) and IgM^+^CD21^hi^ cells (D), as gated in A and B. **(A–D)** *n* = 3. Each circle in the graphs represents data from one animal. Data are representative of three independent experiments. Mean and SD are indicated by horizontal lines in the data points; significance is calculated by one-way ANOVA test (**P < 0.01, ****P < 0.0001). **(E)** The Venn diagram represents the overlap of in vitro OP9-Dll1 culture and primary MZ B cell datasets of the differentially expressed genes between *β1*^*KO*^ and *β1*^*WT*^ cells. The up- and downregulated genes are grouped separately for each comparison. A 1.5-fold change was used as a cutoff for primary cells and a twofold change for in vitro data. **(F)** The overlap of differentially expressed genes in the comparison of *β1*^*KO*^ and *β1*^*WT*^ MZ B cell with the upregulated genes in *β1*^*KO*^ cells relative to *β1*^*WT*^ cells cultured in the presence of OP9-Dll1 cells. The differentially expressed genes in MZ B cells are ranked (y axis) according to the fold-change (x axis). The genes upregulated in the in vitro OP9-Dll1 culture are indicated (right, red). The key genes of interest are highlighted in the figure. RNA-seq was performed once.

**Figure S4.**
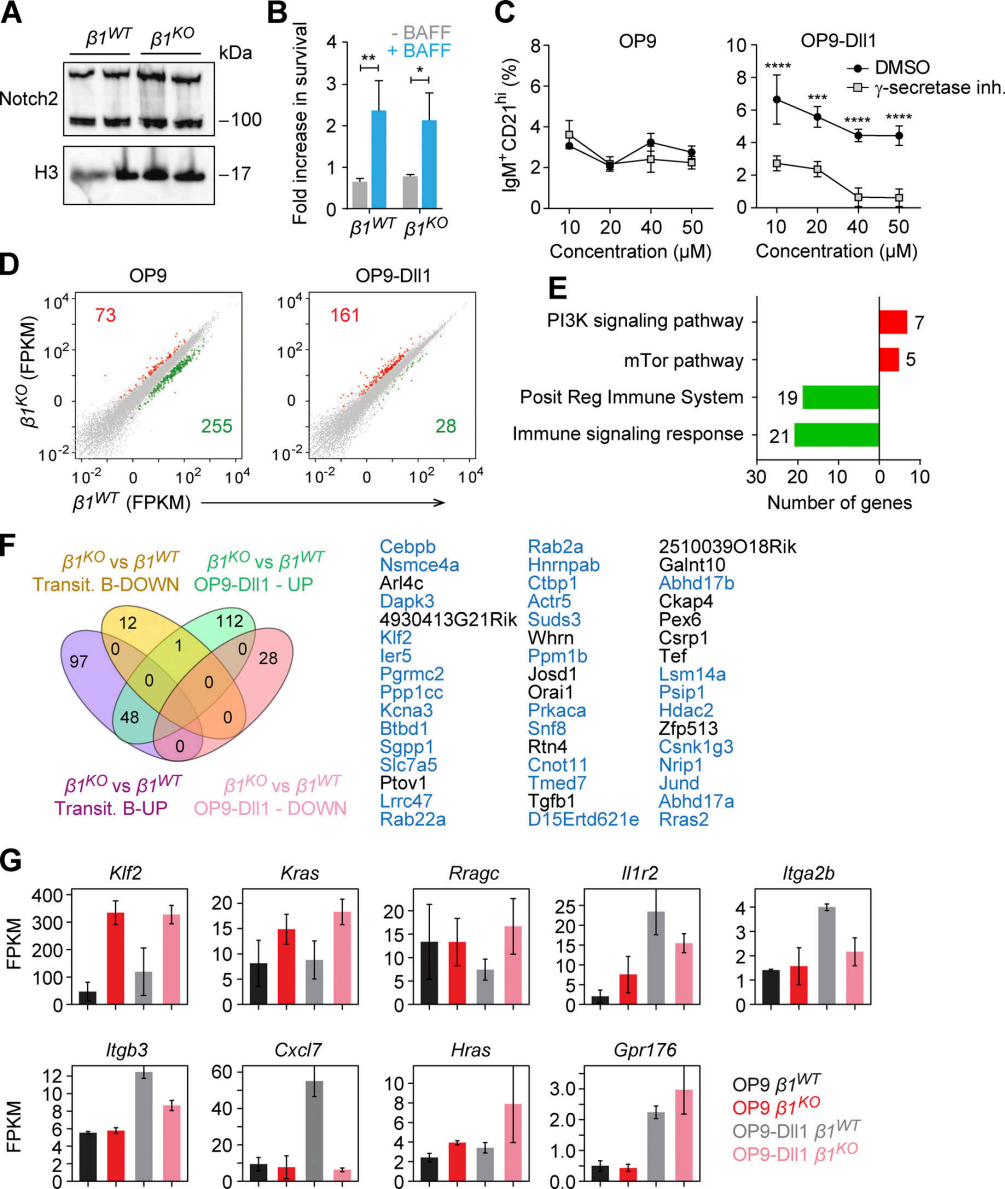
***β1***^***KO***^ **transitional B cells show normal survival and Notch2 expression but different transcriptional profile during differentiation.** Related to Fig. 5. **(A)** Western blot to show Notch2 expression in transitional B cells from *β1*^*WT*^ and *β1*^*KO*^ mice. H3 expression was used as a loading control. Each line represents a different mouse. **(B)** Fold-change in survival of *β1*^*WT*^ and *β1*^*KO*^ transitional B cells, cultured in OP9-Dll1 for 72 h in the absence or presence of the survival factor BAFF. **(C)** Mean (±SD) frequencies of IgM^+^CD21^hi^ cells cultured in OP9 (left panel) and in OP9-Dll1 (right panel) cells for 3 d, with different indicated concentrations of γ-secretase inhibitor or with DMSO (vehicle control). **(A–C)** *n* = 3 mice. Data are representative of at least three experiments; significance calculated by unpaired Student’s *t* test (*P < 0.05, **P < 0.01, *** P < 0.001, ****P < 0.0001). **(D)** Scatter plots represent gene-expression levels of *β1*^*WT*^ (x axis) and *β1*^*KO*^ (y axis) transitional B cells cultured in OP9 (left) and OP9-Dll1 (right). The unaltered (gray), up- (red), and downregulated (green) genes are highlighted. **(E)** Functional classification of up- (red) and downregulated (green) genes in *β1*^*KO*^ transitional B cells cultured in OP9-Dll1 relative to OP9 stromal cells. Numbers next to the bars indicate number of genes associated with each functional class. **(F)** The Venn diagram (left) represents the overlap of in vitro OP9-Dll1 culture and primary transitional B cell datasets of the differentially expressed genes between *β1*^*KO*^ and *β1*^*WT*^ cells. The up- and downregulated genes are grouped separately for each comparison. A 1.5-fold change was used as a cutoff for primary cells and a twofold change for in vitro data. List of the 48 upregulated genes (right) represented in the Venn diagram. Those genes that also overlap between the in vitro OP9-Dll1 culture and primary MZ B cell datasets are highlighted in blue. **(G)** Expression levels (FPKM) of differentially expressed key genes in *β1*^*WT*^ and *β1*^*KO*^ transitional B cells cultured in OP9 and OP9-Dll1 cells. Error bars indicate SD; *n* = 2. RNA-seq was performed once. Source data are available for this figure: SourceData FS4.

As we detected overlapping changes in the expression of genes in MZ B cells and transitional B cells, we further examined whether MZ B cell differentiation is affected by the β1-integrin deficiency. Notch signaling, mediated by the interaction of Notch2 with its ligand Dll1, is required for MZ B cell differentiation (Tanigaki et al., 2002; Saito et al., 2003). Therefore, we cocultured CD93^+^ transitional splenic B cells with either OP9 or OP9-Dll1 stromal cells and added BAFF to enhance cell survival. MZ B cells can be detected by their surface expression of CD1d^hi^CD21^hi^ (Roark et al., 1998) or IgM^+^CD21^hi^ (Martin and Kearney, 2002). Therefore, we used both sets of surface markers to detect MZ B cells by flow cytometric analysis after 72 h of coculture. Coculture of *β1*^*WT*^ transitional B cells with OP9 and OP9-Dll1 cells generated CD1d^hi^CD21^hi^ cells at 3.3 and 23.2% frequencies, respectively (Fig. 5 A). In contrast, coculture of *β1*^*KO*^ transitional B cells with OP9-Dll1 stromal cells increased the CD1d^hi^CD21^hi^ cell population only to 10.8%, which was also reflected by reduced absolute cell numbers relative to *β1*^*WT*^ cocultures (Fig. 5 C). A similar result was obtained by analyzing IgM^+^CD21^hi^ cells. Cocultures of *β1*^*WT*^ transitional B cells with OP9 and OP9-Dll1 stromal cells generated 6.14 and 25.6% IgM^+^CD21^hi^ cells, respectively. However, cocultures of *β1*^*KO*^ transitional B cells with OP9-Dll1 cells generated only 12% IgM^+^CD21^hi^ cells (Fig. 5, B and D). We confirmed that these differences in MZ B cell differentiation were not due to an altered expression of Notch2 or impaired BAFF-mediated survival of *β1*^*KO*^ transitional B cells (Fig. S4, A and B). Moreover, the generation of IgM^+^CD21^hi^ MZ B cells was due to Notch2 signaling since the frequencies of these cells were reduced by adding increasing concentrations of a γ-secretase inhibitor to the OP9-Dll1 cocultures (Fig. S4 C). To better understand the impaired in vitro differentiation of β1-integrin–deficient transitional B cells, we analyzed the transcriptome of *β1*^*KO*^ and *β*1^WT^ transitional B cells that were cocultured with OP9-Dll1 stromal cells. In *β1*^*KO*^ cells, we identified 161 and 28 genes that were up- and downregulated relative to *β1*^*WT*^ cells, respectively (Fig. S4 D). We also observed an enhanced PI3K signature in β1-integrin–deficient in vitro–cultured transitional B cells and a diminished signature for regulation of immune response (Fig. S4 E). By overlapping genes that were deregulated in in vitro–differentiated *β1*^*KO*^ transitional B cells and in ex vivo–sorted *β1*^*KO*^ MZ B cells, we identified 50 genes that were upregulated in both mutant cell populations (Fig. 5 E). This shared set includes genes encoding proteins of the Ras family (*Rras2*, *Rab22a*, *Rab2a*, *Rragc*, and *Kras*), as well as the transcription factor gene *Klf2* and the large neutral amino acids transporter gene *Slc7a5* (Fig. 5 F). Moreover, 48 genes, including *Klf2*, *Slc7a5*, *Rab22a*, and *Rab2a*, were upregulated in both in vitro–cultured *β1*^*KO*^ transitional B cells and ex vivo–sorted *β1*^*KO*^ transitional B cells (Fig. S4 F).

In addition, we compared the transcriptome of *β1*^*KO*^ and *β1*^*WT*^ transitional B cells that were cocultured with OP9 stromal cells and found that *Klf2*, *Kras*, and *Hras* were upregulated independently of Notch signals (Fig. S4 G). In addition, we found that genes encoding the surface receptor IL1R2, the integrins ITGA2B and ITGB3, and the chemokine CXCL7 were downregulated specifically in OP9-Dll1–cocultured *β1*^*KO*^ transitional B cells (Fig. S4 G). Taken together, these transcriptome analyses indicate that the β1-integrin deficiency results in upregulation of genes encoding regulators of MZ B cell differentiation and/or function, whereby the transcriptional changes are further enhanced during Notch-induced differentiation.

**Figure 6.**
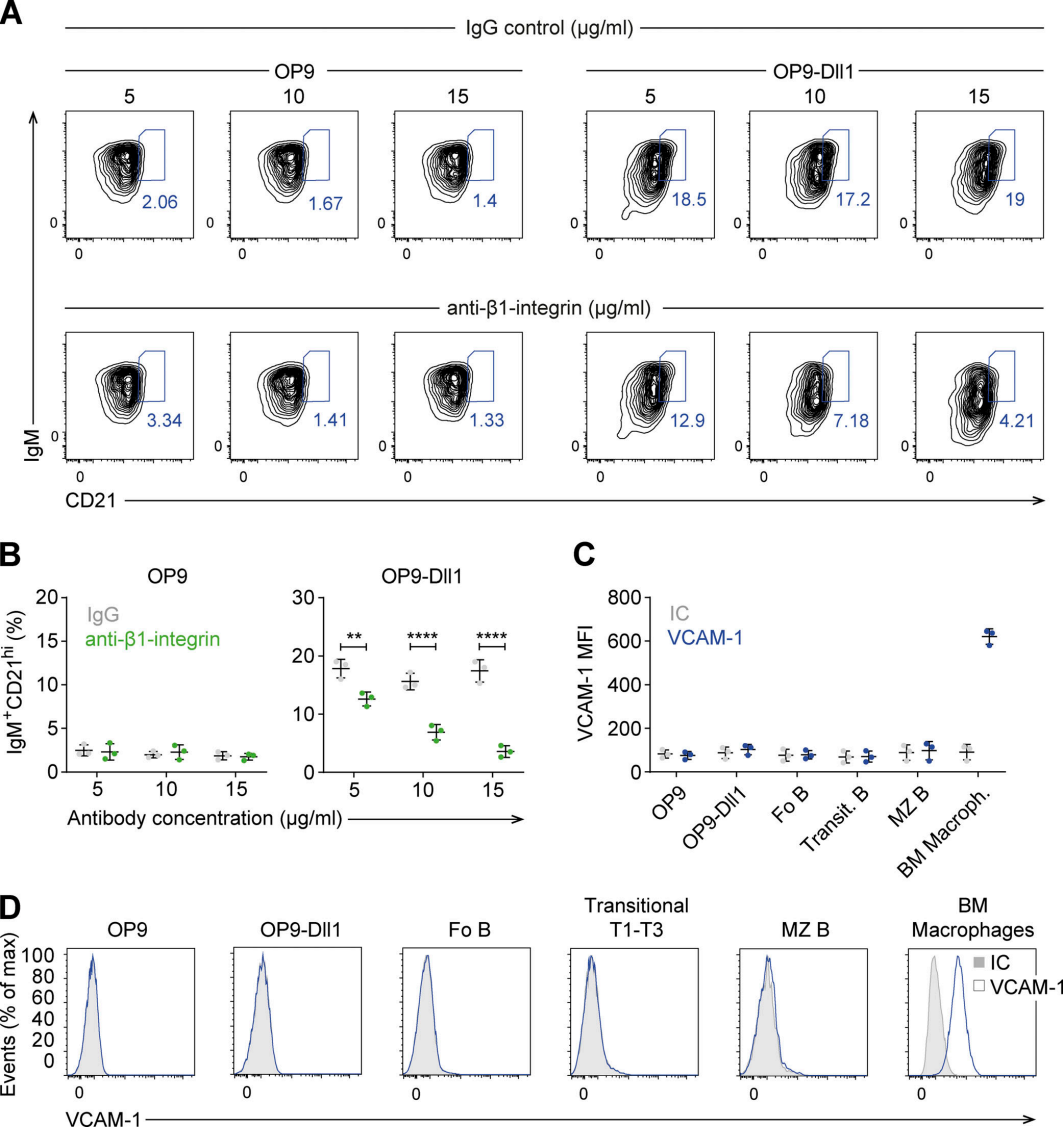
**β1-integrin effect is cell intrinsic. (A)** Flow cytometry to identify the induction of CD21 and IgM expression on transitional B cells from *β1*^*WT*^ mice that were incubated for 30 min with different concentrations of a blocking anti–β1-integrin antibody or with the corresponding IgG control antibody, and later cultured on OP9 or OP9-Dll1 cells for 3 d. **(B)** Mean (±SD) frequencies of the increase in IgM^+^CD21^hi^ cells, as gated in A. **(C and D)** Quantification (MFI; C) and representative histograms (D) of VCAM-1 expression in OP9 and OP9-Dll1 stromal cells, Fo, transitional, and MZ B cells. BM macrophages were used as positive control. IC, isotype control. **(A–D)** *n* = 3. Each circle in the graphs represents data from one mouse. Data are representative of three independent experiments. Mean and SD are indicated by horizontal lines in the data points; significance is calculated by one-way ANOVA test (**P < 0.01, ****P < 0.0001).

To confirm that the effect observed in the in vitro differentiation culture was due to the β1-integrin expression on transitional B cells, and not to an extrinsic effect, we blocked β1-integrin on WT cells by the addition of increasing concentrations of a β1-integrin blocking antibody prior to coculture on OP9 or OP9-Dll1 stromal cells. After 72 h of coculture, we examined the frequencies of IgM^+^CD21^hi^ cells and found that the exposure of WT transitional B cells to anti-β1 antibody reduced the generation of IgM^+^CD21^hi^ MZ B cells in multiple OP9-DLL1 cocultures (Fig. 6, A and B). No significant effects were detected with an isotype control antibody or with the anti-β1 antibody in OP9 cocultures. Thus, treatment of WT transitional B cells with a β1-integrin–blocking antibody had a similar effect as the deletion of the β1-integrin gene.

Considering that integrins are activated through interaction with their ligands on other cells or in the extracellular matrix (Kinashi, 2005), we examined whether an integrin–ligand interaction affects the differentiation of transitional B cells in our in vitro system. Therefore, we analyzed the expression of the main β1-integrin ligand, VCAM-1, in OP9 and OP9-Dll1 stromal cells and in WT primary transitional B, Fo B, and MZ B cells. In none of these cells, we detected VCAM-1 expression (Fig. 6, C and D). As a control and as expected (Chow et al., 2013), abundant VCAM-1 expression was detected in BM macrophages. Although these results suggest that the effects observed in the in vitro differentiation culture do not depend on the integrin–ligand interaction, we cannot rule out that another β1-integrin ligand is expressed on the stromal cells.

### PI3K pathway inhibition in *β1*^*KO*^ transitional B cells enhances MZ B cell differentiation

**Figure S5.**
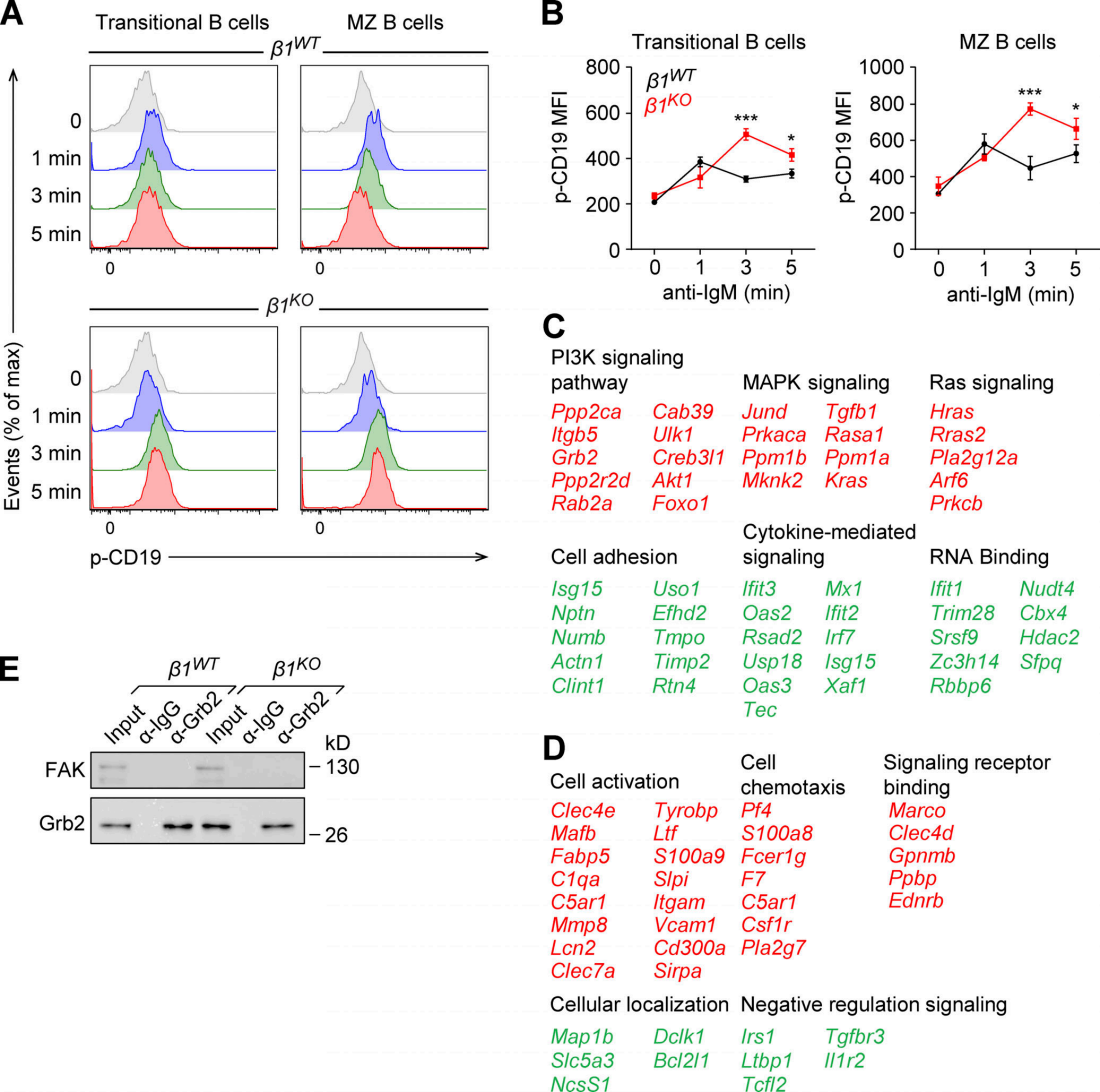
**Increased p-CD19 levels in *****β1**^**KO**^*** transitional and MZ B cells and list of deregulated genes in transitional B cells treated with PI3K** **inhibitor.** Related to Figs. 7 and 8. **(A)** Transitional and MZ B cells from *β1*^*WT*^ and *β1*^*KO*^ mice were stimulated for different time points with anti-mouse IgM F(ab′)2 antibody and the phosphorylation of CD19 was evaluated by flow cytometry. **(B)** Mean (±SD) of phosphorylation of CD19 MFI is plotted. **(A and B)** *n* = 3 mice. Data are representative of three different experiments; significance is calculated by ANOVA test (* P < 0.05, *** P < 0.001). **(C and D)** Selected list of key genes from functional classification of up- (red) and downregulated (green) genes in PI3K inhibitor–treated *β1*^*WT*^ (C) and *β1*^*KO*^ (D) transitional B cells. RNA-seq was performed once. **(E)** Lysates of transitional *β1*^*WT*^ and *β1*^*KO*^ B cells were incubated with beads cross-linked with α-Grb2 or control α-Ig antibodies. Samples were washed and resolved by SDS-PAGE. Grb2 and FAK were detected by immunoblot analysis with specific antibodies. Data are representative of two independent experiments. Source data are available for this figure: SourceData FS5.

**Figure 7.**
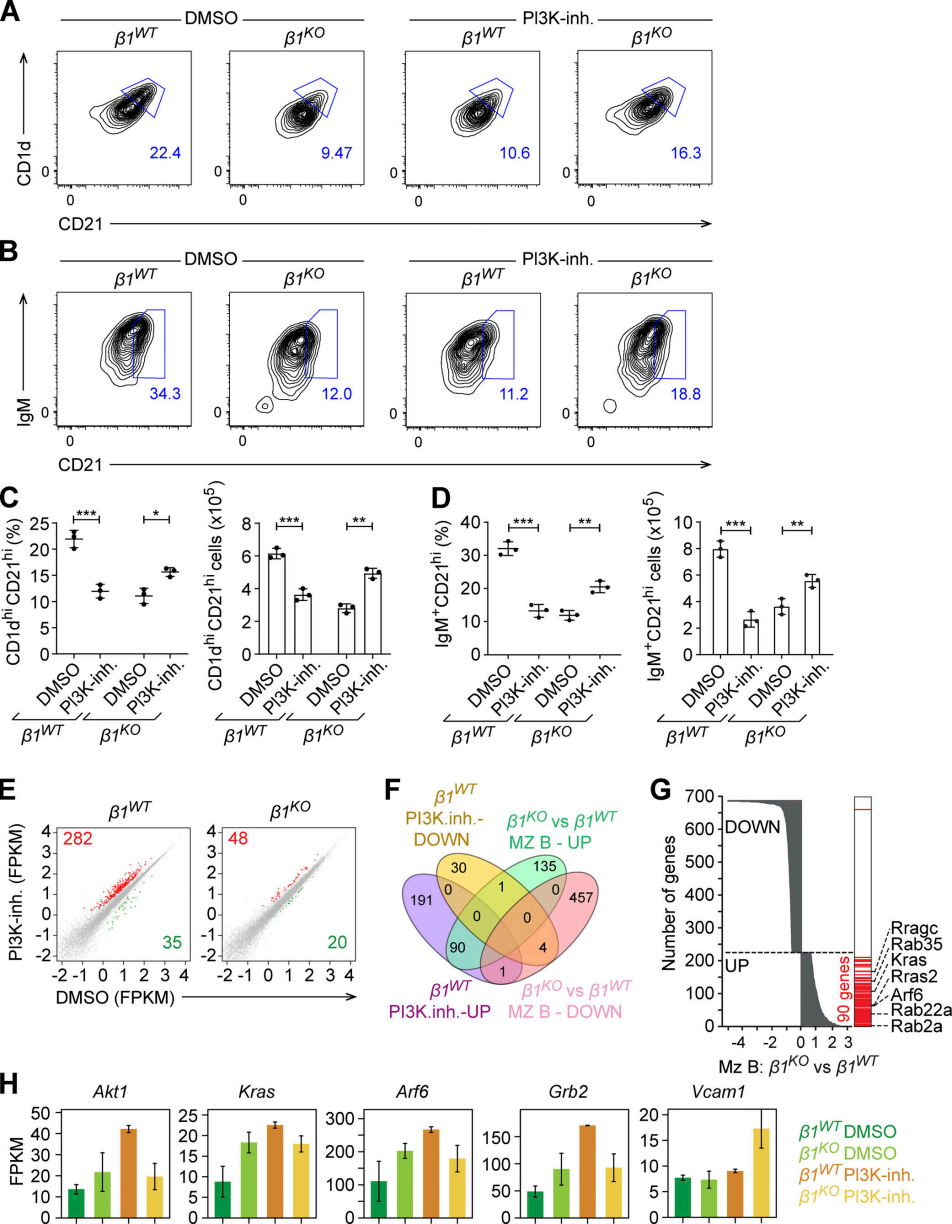
**PI3K pathway inhibition partially rescues the *β1***^***KO***^ **MZ B cell phenotype. (A and B)** Flow cytometry to identify the induction of CD1d and CD21 (A) and IgM and CD21 (B) expression on transitional B cells from *β1*^*WT*^ and *β1*^*KO*^ mice, cultured on OP9 or OP9-Dll1 cells for 3 d in the absence (DMSO) or presence of the PI3K inhibitor (PI3K-inh.). **(C and D)** Mean (±SD) frequencies (upper panel) and absolute numbers (lower panel) of the increase in CD1d^hi^ CD21^hi^ (C) and IgM^+^CD21^hi^ cells (D), as gated in A and B. **(A–D)** *n* = 3 mice. Each circle in the graphs represents data from one mouse. Data are representative of three independent experiments. Mean and SD are indicated by horizontal lines in the data points; significance is calculated by one-way ANOVA test (*P < 0.05, **P < 0.01, ***P < 0.001). **(E)** Scatter plots depict the gene-expression levels in DMSO-treated (x axis) and PI3K inhibitor–treated (y axis) *β1*^*WT*^ (left panel) and *β1*^*KO*^ (right panel) transitional B cells, cultured in OP9-Dll1 cells, as described in A and B. The unaltered (gray), up- (red), and downregulated (green) genes are highlighted. **(F)** The Venn diagram represents the overlap of the differentially expressed genes in the primary MZ B cells with the differentially expressed genes in OP9-Dll1 culture of WT cells treated with PI3K-inh. or DMSO. The up- and downregulated genes are grouped separately for each comparison. A 1.5-fold change was used as a cutoff for primary cells and a twofold change for in vitro data. **(G)** The overlap of differentially expressed genes in the comparison of *β1*^*KO*^ and *β1*^*WT*^ MZ B cells with the upregulated genes in the comparison of OP9-Dll1 culture of WT cells treated with PI3K inhibitor or DMSO. The differentially expressed genes in MZ B cells are ranked (y axis) according to the fold-change (x axis). The genes upregulated after treatment with PI3K inhibitor are indicated (right, red). The key genes of interest are highlighted in the figure. **(H)** Expression levels (FPKM) of differentially expressed key genes in DMSO or PI3K-inh.–treated transitional *β1*^*WT*^ and *β1*^*KO*^ B cells, cultured in the presence of OP9-Dll1 cells, as described in A and B. Error bars indicates SD; *n* = 2.

BCR ligation leads to the Lyn-mediated phosphorylation of the cytoplasmic tail of CD19, which provides binding sites to other kinases, including PI3K (Kurosaki, 2002). By flow cytometry, we observed that the phosphorylation of CD19 after BCR activation is increased in *β1*^*KO*^ transitional and MZ B cells relative to *β1*^*WT*^ cells (Fig. S5, A and B). These results together with our previous data and the described importance of PI3K in the development and activation of mature B cells (Anzelon et al., 2003; Srinivasan et al., 2009; Setz et al., 2018) raised the question of whether the inhibition of PI3K in *β1*^*KO*^ transitional B cells would restore their capacity to differentiate toward a MZ B cell phenotype. To this end, we treated *β1*^*WT*^ and *β1*^*KO*^ CD93^+^ transitional B cells with either the PI3K inhibitor LY294002 or with the vehicle control (DMSO). After 72 h of culturing the treated cells on OP9-Dll1 stromal cells, we analyzed the surface expression of the MZ B cell markers CD1d^hi^CD21^hi^ and IgM^+^CD21^hi^ by flow cytometry. As expected from our previous results, DMSO control-treated *β1*^*KO*^ cell cultures showed a significant decrease in the frequencies and absolute numbers of CD1d^hi^CD21^hi^ cells relative to DMSO control-treated *β1*^*WT*^ cell cultures (Fig. 7, A and C). Interestingly, the treatment of *β1*^*WT*^ cell cultures with the PI3K inhibitor mimicked the decrease of the CD1d^hi^CD21^hi^ cell population observed in DMSO control–treated *β1*^*KO*^ cells. In contrast, PI3K inhibitor–treated *β1*^*KO*^ cell cultures showed a modest but significant increase in the frequencies and numbers of CD1d^hi^CD21^hi^ cells, suggesting a partial rescue of MZ B cell differentiation (Fig. 7, A and C). A similar result was observed when we analyzed the frequencies and numbers of IgM^+^CD21^hi^ cells (Fig. 7, B and D).

Genome-wide RNA-seq analysis of PI3K inhibitor– versus DMSO control–treated transitional B cells showed that 282 genes were upregulated and 35 downregulated in PI3K inhibitor–treated *β1*^*WT*^ cells relative to DMSO control–treated *β1*^*WT*^ cells (Fig. 7 E). In contrast, a total of 68 genes were deregulated in PI3K inhibitor–treated *β1*^*KO*^ cells relative to DMSO-treated *β1*^*KO*^ cells (Fig. 7 E). Bioinformatic Gene Ontology analysis assigned 23 genes that were upregulated in PI3K inhibitor–treated *β1*^*WT*^ transitional B cells to the categories of PI3K signaling (*Grb2*, *Akt1*), MAPK (*Kras*, *Rasa1*) and Ras signaling (*Arf6*, *Hras*, and *Rras2*; Fig. S5 C). For PI3K inhibitor–treated *β1*^*KO*^ cells, the upregulated genes were grouped in the categories of cell activation (*Vcam1*, *C1qa*, and *C5ar1*), cell chemotaxis (*Ccr6*, *Ccl5*), and signaling receptor binding (*Cd28*; Fig. S5 D). Moreover, the overlap of deregulated genes in ex vivo–sorted *β1*^*KO*^ MZ B cells and PI3K inhibitor–treated *β1*^*WT*^ transitional B cells indicated that 90 genes were upregulated in both cell populations (Fig. 7 F). Notably, this set includes genes encoding proteins of the Ras family (*Rab35*, *Rras2*, *Rab22a*, *Rab2a*, *Rragc*, *Kras*, *Arf6*; Fig. 7 G) and were also identified as upregulated genes in the transcriptome of OP9-Dll1–cocultured *β1*^*KO*^ transitional B cells (Fig. 5 F). Gene-specific analysis indicated that the inhibition of the PI3K pathway in *β1*^*WT*^ cells resulted in a similar or greater upregulation of Ras family genes than that observed in DMSO control–treated *β1*^*KO*^ cells (Fig. 7 H). The PI3K-inhibitor treatment of *β1*^*KO*^ cells did not result in a significant change in the expression of Ras family genes relative to DMSO control–treated *β1*^*KO*^ cells, whereas other genes, including *Vcam1*, *Cd28*, *Ccr5*, and *Ccl5*, were upregulated specifically in PI3K inhibitor–treated *β1*^*KO*^ cells (Fig. 7 H). Together, this analysis indicated that the inhibition of the PI3K pathway and the deletion of β1-integrin have similar effects on the expression of Ras family genes. Moreover, the combination of both deficiencies by the PI3K-inhibitor treatment of *β1*^*KO*^ cells results in distinct changes in gene expression that may be linked to the partial rescue of the MZ B cell differentiation defect.

### The adaptor protein Grb2 interacts with the integrin-linked kinase (ILK) in *β1*^*KO*^ B cells

**Figure 8.**
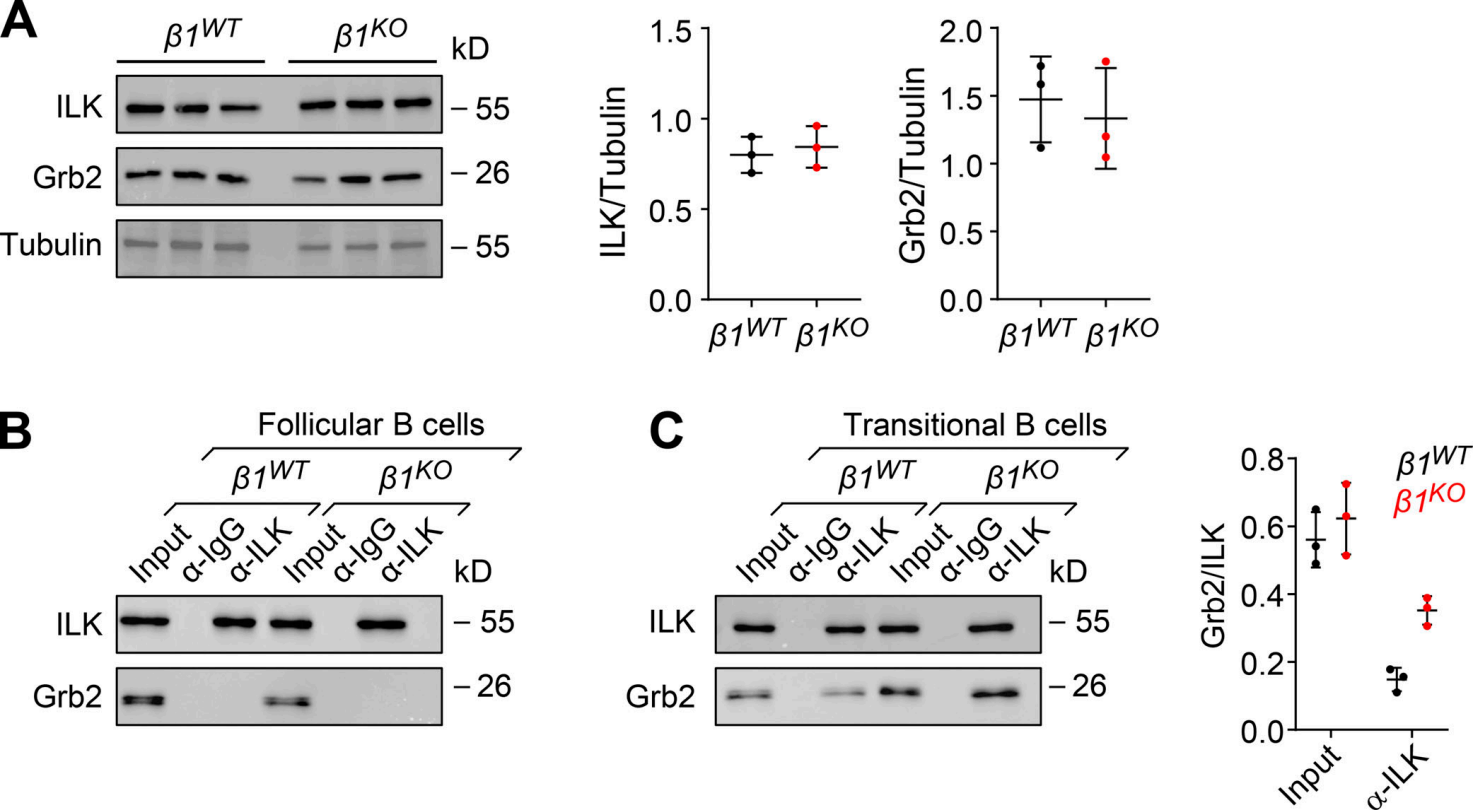
**Grb2 adaptor interacts with ILK in *β1***^***KO***^ **transitional B cells. (A)** Analysis of the expression by Western blot of ILK and Grb2 in *β1*^*WT*^ and *β1*^*KO*^ transitional B cells. Tubulin expression was used as a loading control. Each line represents a different mouse. A quantification of ILK (left) and Grb2 (right)/loading control ratio after normalization is shown for independently performed experiments. The mean ± SD is plotted. Co-IP to detect the association of Grb2 with ILK. **(B and C)** Lysates of *β1*^*WT*^ and *β1*^*KO*^ Fo (B) and transitional (C) B cells were incubated with beads cross-linked with α-ILK or control α-Ig antibodies. Samples were washed and resolved by SDS/PAGE. Grb2 and ILK were detected by immunoblot analysis with specific antibodies. A quantification of the Grb2/ILK ratio for transitional B cells is shown for independently performed experiments. Co-IPs with α-ILK and control α-Ig antibodies are represented by black and red dots, respectively. The mean ± SD is plotted. **(A–C)** *n* = 3 mice. Each circle in the graphs represents data from one mouse. Data are representative of three independent experiments. Mean and SD are indicated by horizontal lines in the data points. Source data are available for this figure: SourceData F8.

Integrins lack an intrinsic enzymatic activity, and therefore their signaling depends on the recruitment of adaptor and signaling proteins (Moser et al., 2009). In particular, ILK has been described as one of these interacting proteins that are recruited to β1- and β3-integrin assembled adhesomes (Schiller et al., 2013) and functions as a scaffold in forming multiprotein complexes that connect integrins to the actin cytoskeleton and to signaling pathways (Legate et al., 2006; Böttcher et al., 2009; Lange et al., 2009). Moreover, integrins can be regulated and activated by different Ras-GTPases in multiple biological contexts (Kinbara et al., 2003). Most upregulated genes in PI3K inhibitor–treated *β1*^*WT*^ cells and DMSO control–treated *β1*^*KO*^ cells encode Ras-GTPase proteins, and therefore, we examined the expression of Grb2, an adapter protein that connects indirectly the IgM-BCR and directly the IgG-BCR to Ras-MAPK activation in B cells (Jang et al., 2009; Engels et al., 2009). First, we evaluated the expression of ILK and Grb2 in *β1*^*WT*^ and *β1*^*KO*^ transitional B cells. Although *β1*^*KO*^ cells have modestly increased levels of Grb2 transcripts (Fig. 7 H), the immunoblot analysis indicated that the protein levels of Grb2 and ILK in *β1*^*KO*^ cells are not altered relative to *β1*^*WT*^ cells (Fig. 8 A). To gain some insight into the mechanism by which the absence of β1-integrin could affect BCR signaling, we examined whether ILK interacts with Grb2. To this end, we performed coimmunoprecipitation (co-IP) of lysates of *β1*^*WT*^ and *β1*^*KO*^ Fo and transitional B cells with anti-Grb2 antibody, followed by immunoblot analysis to detect Grb2 and ILK. No association of these proteins was detected in lysates of *β1*^*WT*^ and *β1*^*KO*^ Fo B cells (Fig. 8 B). However, in lysates of *β1*^*KO*^ transitional B cells, we detected a weak but well above background interaction between ILK and Grb2, which was not observed in lysate of *β1*^*WT*^ cells (Fig. 8 C). As a control of specificity, no interaction of Grb2 with the focal adhesion kinase (FAK), which also associates with β1-integrin, was observed in transitional B cells (Fig. S5 E). Taken together, these data indicate that the impaired MZ B cell differentiation in the absence of β1-integrin may be accounted for by altered interactions of ILK with the BCR signaling regulator Grb2.

## Discussion

The role of α4β1 and αLβ2 integrins in MZ B cells has been associated with cell adhesion and migration, enabling these cells to be retained in the marginal zone of the spleen (Lu and Cyster, 2002). In the present study, we show that β1-integrin has an additional function in the differentiation of transitional B cells to MZ B cells, that is, related to the attenuation of BCR signaling. Differentiation of MZ B cell requires a weaker IgM-BCR signaling response than differentiation of Fo B cells (Pillai and Cariapa, 2009; Cerutti et al., 2013), and we find that β1-integrin deficiency in B cells results in enhanced BCR signaling, Ca^2+^ mobilization, and Erk activation. The β1-integrin deficiency also results in the upregulation of genes related to the Ras/MAPK signaling, similar to the pharmacological inhibition of the PI3K pathway in WT B cells. In β1-integrin–deficient B cells, we detect an interaction of the adaptor protein Grb2 with ILK, raising the possibility that an altered β1-integrin expression during MZ B cell differentiation confers a dampened B cell signaling response via Grb2.

The MZ B cell defect in β1-integrin–deficient mice and cell cultures differs from a previous study in which β1-integrin function was evaluated in murine BM chimeras (Brakebusch et al., 2002). However, in the BM chimeras, 15% of the splenic cells still expressed β1-integrin, and the analysis of the MZ B cells included other splenic B cell populations (such as transitional B cells), which could have affected the analysis. Our B cell–specific deletion of a single integrin gene, *Itgb1*, did not result in a detectable mobilization of MZ B cells from the spleen to peripheral blood, which has been observed in mice with impaired function of multiple integrins. These studies included mice treated with α4β1- and αLβ2-blocking antibodies (Lu and Cyster, 2002); mice with a deletion of the *Kindlin 3* gene (Härzschel et al., 2021), encoding an activator of β1-, β2-, and β3-integrins (Moser et al., 2009); and mice with a B cell–specific deletion of the chaperone Grp94, which affects the folding of multiple integrins (Staron et al., 2010). The lack of accumulation of MZ B cells in peripheral blood of the β1-integrin–deficient mice is consistent with a recent analysis of α4-integrin–deficient mice, which have a reduced MZ B cell population in the spleen but no mobilization of these cells to the periphery (Härzschel et al., 2021). These data suggest that the deletion of a single integrin may not be sufficient to cause an adhesion defect in the spleen, possibly because of compensation by other integrins. However, we cannot rule out a transient release of β1-deficient MZ B cells from the spleen as we used a chronic (genetic) deletion of the β1-integrin gene by Cd21-Cre or Cd19-Cre. By a tamoxifen-induced deletion of the *Kindlin 3* gene, the release of MZ B cells has been detected at 1 wk but not at 2 wk after deletion (Härzschel et al., 2021).

The reduced accumulation of MZ B cells in the spleen, despite the lack of obvious proliferation or survival defects on these cells and their precursors, the transitional B cells, can be accounted for by the differentiation defect of *β1*^*KO*^ transitional B cells to MZ B cells in vitro. One of the signals required for MZ B cell development and the maintenance of MZ B cell identity is the activation of the Notch2 receptor by the Dll1 ligand, which is expressed on stromal cells (Tanigaki et al., 2002; Saito et al., 2003; Hozumi et al., 2004; Tan et al., 2009). By using an in vitro coculture of *β1*^*KO*^ transitional B cells with OP9-Dll1 stromal cells, we observed a reduced surface expression of CD1d, IgM, and CD21 relative to *β1*^*WT*^ cell cultures. These differences were likely cell-intrinsic because neither the OP9 stromal cells nor the transitional B cells express the major β1-integrin ligand, VCAM-1. Moreover, the effects were independent of the addition of Mg^2+^ or Mn^2+^ to our culture medium, which is known to generally augment integrin activation (Lenter et al., 1993; Ye et al., 2012). However, the addition of β1-integrin–blocking antibody to WT transitional B cells resulted in a similar differentiation defect as the genetic deletion of the β1-integrin gene, and therefore, we cannot exclude the possibility that another β1-integrin–activating ligand is present on the OP9 stromal cells.

Moreover, *β1*^*KO*^ transitional B and MZ B cells show overlapping and distinct changes in their transcriptomes, relative to their WT counterparts. In particular, β1-deficient MZ B cells show an upregulation of the *Klf2* gene, encoding a transcription factor that is part of a regulatory network distinguishing MZ B and Fo B cells (Hoek et al., 2010; Hart et al., 2011; Winkelmann et al., 2011). *Klf2* is downregulated during differentiation of transitional B to MZ B cells, and the knockout of *Klf2* results in enhanced MZ B cell differentiation (Hoek et al., 2010; Hart et al., 2011; Winkelmann et al., 2011). In addition, *Klf2* inactivation results in an increase in Igλ-expressing B cells, correlating with the elimination of autoreactive B cells (Hart et al., 2011). Both phenotypes correlate inversely with those of the β1-integrin deletion, raising the possibility of a functional relationship between Klf2 and β1-integrin. Although *Foxo1*, whose genetic deletion induces an expansion of the MZ B cell population (Chen et al., 2010), was upregulated in *β1*^*KO*^ transitional B cells, we did not observe a Foxo1-associated gene signature in the mutant cells. Instead, we detected an enhanced mTORC1 signature, defined by an increase in the gene sets associated with cell proliferation (G2M checkpoint, E2F targets, and Myc targets) and activation (IL2-STAT5 signaling and TNFα signaling via NF-κB; Sintes et al., 2017). Thus, the PI3K–Akt–mTORC1 signaling axis may be altered in the absence of β1-integrin. Interestingly, mTORC1 signaling has been recently associated with the enhanced transcription of unfolded protein response–related genes and rapid division-independent PC differentiation of MZ B cells (Gaudette et al., 2020; Gaudette et al., 2021).

The role of β1-integrin was not limited to MZ B cell development but included PC differentiation upon immunization with a TI antigen. In immunized *β1*^*KO*^ mice, we observed a reduction of serum IgM and IgG3 levels, as well as low frequencies and absolute numbers of splenic PCs. The diminished PC population in *β1*^*KO*^ mice could in principle be accounted for by the reduction of the MZ B cell population, which is the main source of PCs in a TI immune response that involves crosstalks with innate lymphoid cells in the marginal zone of the spleen (Cerutti et al., 2013; Magri et al., 2014). However, in an LPS-induced differentiation of *β1*^*KO*^ B220^+^ B cells, in which we used the same number of B220^+^ cells as in parallel *β1*^*WT*^ cultures, we also observed a reduction in the generation of pre-PB and PB, indicating that the impaired TI-dependent *β1*^*KO*^ PC generation cannot be accounted for by reduced precursor cell numbers.

In contrast to the TI immune response, TD antigen-induced PC development, which is primarily mediated by Fo B cells, was not affected in *β1*^*KO*^ mice, consistent with the normal numbers of splenic Fo B cells in these mice. These results are consistent with a previous study of the roles of β1- and β2-integrins in the GC response, in which it was demonstrated that the GC formation upon TD-antigen immunization is not affected by the β1- and β2-integrin deletion (Wang et al., 2014).

Differentiation of transitional B cells toward Fo B or MZ B cells is also regulated by the strength of the B cell signal, with a strong IgM-BCR signal favoring Fo B cells and a weak IgM-BCR signal promoting MZ B cell development (Pillai and Cariapa, 2009; Cerutti et al., 2013). *β1*^*KO*^ transitional B cells showed enhanced IgM-BCR signaling relative to *β1*^*WT*^ cells, as evidenced by the increased phosphorylation of the kinases Lyn and Syk after an anti-IgM treatment. Consistent with the enhanced IgM-BCR signaling of *β1*^*KO*^ cells, the Ca^2+^ flux was also increased in these cells. Moreover, in *β1*^*KO*^ transitional B cells, we observed enhanced phosphorylation of Akt and Erk prior to IgM-BCR stimulation, with a sustained activity over time, suggesting an activation of two different signaling pathways, the mTORC1 and the Ras-controlled Erk/MAP kinase pathway. These data raise the interesting possibility that β1-integrin promotes MZ B cell development by regulating BCR, PI3K, and MAP kinase signaling.

By analyzing the transcriptome of *β1*^*KO*^ transitional B cells and MZ B cells ex vivo and in vitro cultures, we found a striking enrichment of genes associated with Ras-GTPase–related proteins that were upregulated in the mutant cells. Notably, we detected a similar upregulation of these genes in *β1*^*WT*^ transitional B cells that were treated with a pharmacological inhibitor of the PI3K pathway. PI3K has an important role in the development of mature B cells (Srinivasan et al., 2009; Setz et al., 2018) and its levels and activation must be highly regulated to avoid autoimmunity and B cell malignancies (Okkenhaug and Vanhaesebroeck, 2003). A positive correlation between PI3K signaling and MZ B cell differentiation has been reported (Clayton et al., 2002; Anzelon et al., 2003; Durand et al., 2009), consistent with the observed decrease of MZ B cells in the PI3K inhibitor–treated WT transitional B cell. Surprisingly, the addition of the PI3K inhibitor to the *β1*^*KO*^ transitional B cell cultures resulted in a modest but significant increase in the generation of MZ B cells, which may be due to a compensatory crossregulation of the PI3K and Ras pathways (Mendoza et al., 2011).

The adaptor protein Grb2 interacts with the regulatory p85 subunit of PI3K (Wang et al., 1995; Wheeler and Domain, 2001) and with other key activators of PI3K signaling, including CD19 and BCAP (Neumann et al., 2009). Previous studies have shown that Grb2 functions as an inhibitor of the IgM-BCR/PI3K pathway in B cells that regulates B cell activity and B cell differentiation (Manno et al., 2016). Moreover, mice in which the *Grb2* gene has been deleted in B cells have reduced MZ B cell numbers, altered spleen architecture, increased IgM-BCR signaling and Ca^2+^ flux, resembling the phenotypes of *β1*^*KO*^ mice (Jang et al., 2011; Ackermann et al., 2011). Thus, Grb2 may be functionally linked with the altered CD19/PI3K signaling in *β1*^*KO*^ transitional B cells.

The adaptor protein Grb2 encompasses a central SH2 domain that is flanked by two SH3 domains, enabling the interaction with phosphorylated tyrosines and other protein domains. In addition to components of the BCR and PI3K pathways, Grb2 interacts with components of the integrin signaling pathway. In particular, Grb2 has been shown to interact with the FAK (Schlaepfer et al., 1994, 1998), a protein-tyrosine kinase that is activated after integrin binding to extracellular matrix proteins (Mitra et al., 2005). In our analysis, we did not detect an interaction between Grb2 and FAK, probably because this association has been detected only under conditions in which the integrin was activated by plating cells on fibronectin (Schlaepfer et al., 1994; Schlaepfer et al., 1998). In our analysis, however, we evaluated this interaction with primary cells without an activation of integrins. In contrast, we detected an interaction of Grb2 with ILK in β1-integrin–deficient cells. ILK is a central component of the intracellular ILK–pinch–parvin complex that localizes together with paxillin to focal adhesions and regulates integrin-mediated cell functions. First described as a kinase, now it is known that ILK functions as a pseudokinase (Wickström et al., 2010). ILK is recruited to β1- and β3-integrin–containing adhesomes where it binds different substrates, including regulators of small GTPases involved in the regulation of cell survival, proliferation, and migration (Hannigan et al., 1996; Legate et al., 2006). Therefore, we favor the view that β1-integrin augments MZ B cell differentiation via the recruitment of ILK and an altered activity of the BCR signaling adaptor Grb2, leading to an enhancement of the mTORC1 and Erk/MAP kinase pathways. However, we still have limited insight into how enhanced PI3K signaling in β1-integrin–deficient transitional B cells leads to impaired MZ B cell formation. Future experiments will also have to determine the involvement of altered BCR signaling in the mutant phenotype and examine whether the rescue of the MZ B cell defect by inhibition of the PI3K is a result of enhanced BCR signaling or mediated by another mechanism.

## Materials and methods

### Mice

All mouse experiments were carried out in accordance with the guidelines of the Federation of European Laboratory Animal Science Association and following legal approval of the Regierungspräsidium Freiburg. Floxed β1 integrin mice were already described (Potocnik et al., 2000), *Prdm1*^*+/gfp*^ mice were obtained from the laboratory of Stephen Nutt (The Walter and Eliza Hall Institute of Medical Research, Parkville, Australia), and *Igh*^*B1–8*^ mice were from the laboratory of Klaus Rajewsky (Max-Delbrück-Center for Molecular Medicine, Berlin, Germany). All strains were intercrossed with *Cd21*^*Cre*^ transgenic mice, and floxed β1 integrin mice were intercrossed also with *Cd19*^*Cre*^ transgenic mice (Rosenbaum et al., 2014). Mouse strains were bred and maintained in the Max Planck Institute of Immunobiology and Epigenetics Freiburg’s conventional animal care facility. Experiments were performed in 6–12-wk-old mice from C57BL/6J background.

### Flow cytometry

Single-cell suspensions were resuspended in PBS 2% FCS and stained for flow cytometric analysis. Data were acquired with a LSR Fortessa (BD Biosciences) and analyzed using FlowJo software. Antibodies against the following molecules were used: CD19 (6D5), CD93 (AA4.1), CD23 (B3B4), CD1d (1B1), GL7 (GL-7), β1-integrin (HMb1-1), β2-integrin (M18/2), VCAM-1 (429), anti-rat PE to detect VCAM-1, and IgG2a isotype control were from eBioscience; CD21 (7G6), B220 (RA3-6B2), CD138 (281-2), CD5 (53-7.3), IgM (R6-60.2), Igλ (R26-46), Fas (Jo2), Gr1 (RB6-8C5), and α4-integrin (9C10) from BD. αL-integrin (M17/4) and CD11b (M1/70) were from BioLegend. NP hapten conjugated to PE was from Biosearch Technologies. For detecting intracellular phosphorylated proteins, cells were stained for 20 min with surface markers and then cells were fixed and permeabilized with Cytofix/Cytoperm solution according to the protocol’s instructions (catalog No. 554723; BD Biosciences). Cells were incubated overnight (ON) with the unconjugated rabbit anti-mouse p-Syk (C87C1; Cell Signaling Technology) and rabbit anti-mouse p-Lyn (Cell Signaling Technology) followed by AF488 or AF647-conjugated anti-rabbit secondary antibody. Anti-CD16/32 (93; BD) was used to block nonspecific binding.

### Immunizations and ELISA

Mice were injected intraperitoneally with 50 μg TNP-LPS or 150 μg adsorbed NP-KLH (Biosearch Technology) 1:1 ratio onto Alu-Gel-S (Serva). Spleens and BM were taken after the indicated time points p.i., and PCs were analyzed by flow cytometry. Blood samples were taken at the indicated time points p.i. TNP-specific antibodies were detected by ELISA, using TNP-BSA (10 µg/ml) for capture and biotinylated anti-mouse IgM (Southern Biotech), IgG1 (Southern Biotech), and IgG3 (BD Bioscience) for detection. Mouse sera were serially diluted in duplicate with an appropriate standard (mouse a-TNP-IgM; 55581, BD Pharmingen, or a reference sample). ELISA plates were developed with alkaline-phosphatase streptavidin (Sigma-Aldrich) and phosphorylated nitrophenyl phosphate (Sigma-Aldrich). Absorbance at 405 nm was determined with a SPECTRAmax 250 plate reader (Molecular Device).

### Confocal imaging

Confocal imaging was performed on spleen sections. The following antibodies were used: IgM (II/41; Thermo Fischer Scientific), IgD (11-26.c2a; BioLegend), CD1d (1B1; eBioscience), CD169 (MOMA-1; Abcam). Briefly, 8-μm spleen frozen sections were fixed for 10 min in cold acetone. After washing with PBS, sections were blocked with 10% BSA or Streptavidin/Biotin Blocking kit (Vector) in the case of the MOMA-1 biotinylated antibody. After washing with PBS, sections were stained with the primary antibodies ON at 4°C, followed by a 60-min incubation period with Streptavidin-BV-421 (BioLegend) for MOMA-1 antibody. Sections were mounted with ProLong Gold antifade reagent (Thermo Fisher Scientific) and images were acquired on a Zeiss LSM780 confocal microscope equipped with 488-, 561-, and 633-nm lasers. Images were analyzed with Imaris software.

### In vitro differentiation of PBs

To mimic TI immunization in vitro, splenic B cells were purified from *β1*^*WT*^ and *β1*^*KO*^ CD21cre *Prdm1*^*+/gfp*^ mice using anti-B220 magnetic beads (Miltenyi Biotec) and cultured with 25 μg/ml LPS (L5668; Sigma-Aldrich). After 4 d, three populations were differentiated: CD138^−^Blimp^−^ Act B cells, CD138^−^Blimp^+^ (pre-PB), and CD138^+^Blimp^+^ (PB). To differentiate CD138^+^Blimp^+^ cells under TD conditions, B220^+^ cells were cultured for 5 d in the presence of CD40L (5 ng/ml), IL-4, and IL-5 (10 ng/ml; Peprotech).

### Cell cycle analysis and annexin V staining

For cell cycle analysis, splenic cells from *β1*^*WT*^ and *β1*^*KO*^ mice were fixed and permeabilized for 30 min at 4°C with Cytofix/Cytoperm solution, according to protocol’s instructions (catalog No. 554723; BD Biosciences). Subsequently, intracellular Ki67 (eBioscience) staining was performed for 30 min at room temperature, and FxCycle Violet (Thermo Fischer Scientific) at 1 μg/ml was added for 10 min prior to flow cytometry analysis. For apoptosis assay, cells were stained for Annexin V-FITC and 7AAD according to the manufacturer’s guidelines (BD Bioscience). Samples were acquired in LSR Fortessa flow cytometer within 30 min and analyzed with FlowJo software.

### Differentiation of transitional B cells on OP9-Dll1 cells

OP9-Dll1 or control OP9 cells were grown to 80% confluence in 24-well plates in optimized medium (α-MEM supplemented with 20% FCS). For enrichment of transitional B cells, red cell lysis was performed first on spleen suspensions. Cells were washed, incubated first with anti-CD19 magnetic beads, sorted using LS columns, and later incubated with anti-CD93 magnetic beads to be sorted again with LS columns (beads and columns from Miltenyi Biotec). Subsequently, 5 × 10^5^ magnetically enriched CD19^+^CD93^+^ (AA4.1^+^) transitional B cells were added to the OP9 cells in a new optimized medium (IMDM supplemented with 10% FCS). The B cell growth and survival factor BAFF (10 ng/ml; R&D Systems) was added to maintain cell survival, and cells were harvested for flow cytometry analysis 3 d later. Survival was analyzed using flow cytometry adding Fixable Viability dye eFluor 780 (eBioscience) to the cells. For γ-secretase inhibitor experiments, sorted transitional B cells were cultured with vehicle control (DMSO) or with increasing concentrations of γ-secretase inhibitor (DAPT-Abcam) for 72 hr on OP9 and OP9-Dll1 stromal cells as described. For blocking β1-integrin experiments, sorted transitional B cells were incubated with 5, 10, or 15 µg/ml of IgG Isotype Control (eBio299Arm) antibody or CD29 (Integrin β1) Monoclonal Antibody (HMb1-1; both from eBioscience), for 30 min on ice. After incubation, cells were washed with PBS, resuspended in complete IMDM, and cultured on OP9 and OP9-Dll1 stromal cells as described. For the PI3K inhibitor experiments, CD93^+^ (AA4.1^+^) transitional B cells were incubated with DMSO or PI3K inhibitor (10 µM; LY294002; Cell signaling Technology) for 30 min at 37°C. Cells were washed and cultured in OP9 cells as described.

### mRNA preparation and RNA-seq analysis

Total RNA was isolated from FACS-sorted Fo (CD19^+^CD93^−^CD23^+^CD21^−^), transitional (CD19^+^CD93^+^), and MZ (CD19^+^CD93^−^CD23^−^CD21^+^) B cells from *β1*^*WT*^ and *β1*^*KO*^ mice, and from CD93^+^ (AA4.1+) transitional B cells that were incubated with DMSO or PI3K inhibitor (10 µM; LY294002; Cell signaling Technology) using a RNeasy Mini Kit (Qiagen) and treated with DNase I according to the manufacturer’s instructions. The total mRNA was enriched by Oligo-dT magnetic beads. The libraries were prepared by using a TruSeq Stranded mRNA library preparation kit using the NEB Next Low Input RNA Library preparation protocol. The samples were sequenced using Illumina NovaSeq6000. The base calling was performed by using BCL2Fastq pipeline (v0.3.1) and bcl2fastq (v2.20.0.422). The 100 bp paired-end RNA-seq reads were trimmed using trimgalore and mapped to the mouse reference genome (mm10) using STAR (v2.5.3a; Dobin et al., 2013). The mapped reads were further assembled using Cufflinks (v2.2.1), and the expression level of the known annotated genes (UCSC, mm10) was calculated by Cuffquant. The two biological replicates of each condition were normalized and the differential gene expression between the conditions was calculated by using Cufflinks tools (Cuffnorm and Cuffdiff; Trapnell et al., 2012). The differentially expressed genes were filtered with the q-value cut-off <0.05 following Benjamini–Hochberg multiple testing correction of the original P values. The gene sets were further filtered for more than twofold up- or downregulation. The differentially expressed genes were curated using gene ontology, panther functional classifications, mSignatureDB, and based on the published literatures.

### Ca^2+^ flux

For Ca^2+^ flux analysis, cells were labeled in the dark with 5 μg/ml Indo-1 and 0.5 μg/ml Pluronic F-127 (both from Molecular Probes, Life Technologies) for 45 min in RPMI containing 1% FCS. Cells were washed and kept on ice in RPMI 1% FCS until measurement. The baseline was recorded, and cells were stimulated with 5 μg/ml of anti-mouse IgM (Invitrogen). The change of the ratio of Ca^2+^-bound versus Ca^2+^-unbound Indo-1 was followed for 5 min with an LSRIII fluorescence spectrometer (BD). Data were analyzed with FlowJo software.

### Cell stimulation and immunoblot analysis

Cells were starved for 1 h and then stimulated for the indicated times at 37°C with 10 μg/ml anti-mouse IgM F(ab′)2 antibody (Dianova). Stimulation was stopped with cold PBS and cells were lysed for immunoblot analysis with buffer containing 20 mM Hepes (pH 7.6), 2 mM MgCl_2_, 150 mM NaCl, 10% glycerol, 0.1% NP40, 1 mM Na_3_VO_4_, 1 mM PMSF, and protease inhibitor mix (Sigma-Aldrich). Protein extracts corresponding to equal cell numbers were loaded onto the SDS-PAGE gel. The samples were then blotted with the following antibodies to H3 (rabbit polyclonal, Abcam); p-Syk (Tyr525/526; rabbit monoclonal; Cell Signaling Technology); p-Lyn (Tyr507; rabbit polyclonal; Cell Signaling Technology); p-Akt (Ser473; rabbit monoclonal; Cell Signaling Technology); and p-44/42 (Erk1/2; Thr202/Tyr204; rabbit monoclonal; Cell Signaling Technology). For Notch2, ILK, Grb2, and FAK immunoblots, cells were lysed as detailed above and samples were blotted with Notch2 antibody (D76A6; rabbit monoclonal; Cell Signaling Technology), ILK antibody (4G9; rabbit monoclonal; Cell Signaling Technology), and Grb2 antibody (Y237; rabbit monoclonal; Abcam), respectively, and Tubulin-HRP (Mouse IgG2b, Proteintech). Immunoblots were developed with Chemidoc Imaging System (BioRad) and analyzed with Image Lab software (BioRad).

### Co-IP

*β1*^*WT*^ and *β1*^*KO*^ Fo and transitional B cells were lysed with buffer containing 20 mM Hepes (pH 7.6), 2 mM MgCl_2_, 150 mM NaCl, 10% glycerol, 0.1% NP40, 1 mM PMSF, and protease inhibitor mix (Sigma-Aldrich). Following sonication, the cell lysate was centrifuged at 16,100 *g* for 10 min at 4°C. The protein concentration of the supernatant was measured by Bradford assay, and 1 mg of total protein was then mixed with anti-ILK (4G9; rabbit monoclonal; Cell Signaling Technology) or anti-rabbit IgG isotype control antibody (Biomol) and rotated ON at 4°C. Subsequently, samples were incubated with Dynabeads Protein G (Thermos Fischer Scientific) for 2 h at 4°C, beads were washed, and associated proteins were eluted by addition of 2× sample buffer and a boiling step of 10 min at 95°C. Following separation of the proteins by SDS-PAGE, proteins were detected with anti-ILK (4G9; rabbit monoclonal; Cell Signaling Technology), anti-Grb2 (Y237; rabbit monoclonal; Abcam), and anti-FAK antibody (rabbit polyclonal; Cell Signaling Technology).

### Statistics

Data are expressed as mean ± SD, as indicated in the figure legends. Details of statistical tests and the exact replicate numbers are reported in the figure legends. Except for sequencing analysis, all statistical analyses were performed using Prism 8 software (GraphPad).

### Online supplemental material

Fig. S1 shows reduced β1-integrin expression and MZ B cell frequencies in *β1*^*KO*^ mice (data related to Fig. 1). Fig. S2 shows that *β1*^*KO*^ mice have normal PC differentiation in TD immune responses (data related to Fig. 2). Fig. S3 shows that *β1*^*KO*^ MZ B cells have altered self-antigen recognition (data related to Fig. 4). Fig. S4 shows that *β1*^*KO*^ transitional B cells have normal survival and Notch2 expression, but have different transcriptional profiles during differentiation (data related to Fig. 5). Fig. S5 shows p-CD19 levels in transitional B and MZ B cells from *β1*^*WT*^ and *β1*^*KO*^ mice and includes a list of key deregulated genes in PI3K inhibitor–treated *β1*^*KO*^ vs. *β1*^*WT*^ transitional B cells with their functional classification, as well as data of co-IP of Grb2 and FAK in transitional *β1*^*WT*^ and *β1*^*KO*^ B cells (data related to Fig. 7 and Fig. 8). Data S1 includes a list of up- and downregulated genes in *β1*^*KO*^ vs. *β1*^*WT*^ transitional B and MZ B cells and a list of overlapping genes between these cells. Data S2 includes a list of up- and downregulated genes in *β1*^*KO*^ vs. *β1*^*WT*^ transitional B cells, cocultured with OP9-Dll1 stromal cells, as well as a list of deregulated genes in cultured *β1*^*KO*^ vs. *β1*^*WT*^ transitional B cells that overlap with datasets of the differentially expressed genes in *β1*^*KO*^ versus *β1*^*WT*^ primary transitional B cells. Data S3 includes a list of differentially expressed genes in PI3K inhibitor–treated *β1*^*WT*^ vs. *β1*^*KO*^ transitional B cells in OP9-Dll1 coculture, and a list of differentially expressed genes in primary *β1*^*KO*^ vs. *β1*^*WT*^ MZ B cells that overlap with differentially expressed genes in PI3K inhibitor–treated vs. untreated *β1*^*WT*^ cells.

## Supplementary Material

Data S1 includes a list of up- and downregulated genes in *β1*^*KO*^ vs. *β1*^*WT*^ transitional B and MZ B cells and a list of genes that are deregulated in both *β1*^*KO*^ transitional B and *β1*^*KO*^ MZ B cells.

Data S2 includes a list of up- and downregulated genes in *β1*^*KO*^ vs. *β1*^*WT*^ transitional B cells, cocultured with OP9-Dll1 stromal cells, as well as a list of deregulated genes in cultured *β1*^*KO*^ vs. *β1*^*WT*^ transitional B cells that overlap with datasets of the differentially expressed genes in *β1*^*KO*^ versus *β1*^*WT*^ primary transitional B cells.

Data S3 includes a list of differentially expressed genes in PI3K inhibitor–treated *β1*^*WT*^ vs. *β1*^*KO*^ transitional B cells in OP9-Dll1 coculture, and a list of differentially expressed genes in primary *β1*^*KO*^ vs. *β1*^*WT*^ MZ B cells that overlap with differentially expressed genes in PI3K inhibitor–treated vs. untreated *β1*^*WT*^ cells.

SourceData F4 contains original blots for Fig. 4.

SourceData F8 contains original blots for Fig. 8.

SourceData FS4 contains original blots for Fig. S4.

SourceData FS5 contains original blots for Fig. S5.

## Data Availability

Data from RNA-seq have been deposited in Gene Expression Omnibus repository and are available under accession number GSE213512.
