# Supplementary material for: Integrin β1 regulates marginal zone B cell differentiation and PI3K signaling
Source: J Exp Med. 2022 Nov 9;220(1):e20220342. doi: 10.1084/jem.20220342 (PMC9814157; doi:10.1084/jem.20220342)
Supplement: SourceData F4 — contains original blots for Fig. 4. [file JEM_20220342_SourceDataF4.pdf]

Andreani_Source Data_Fig 4

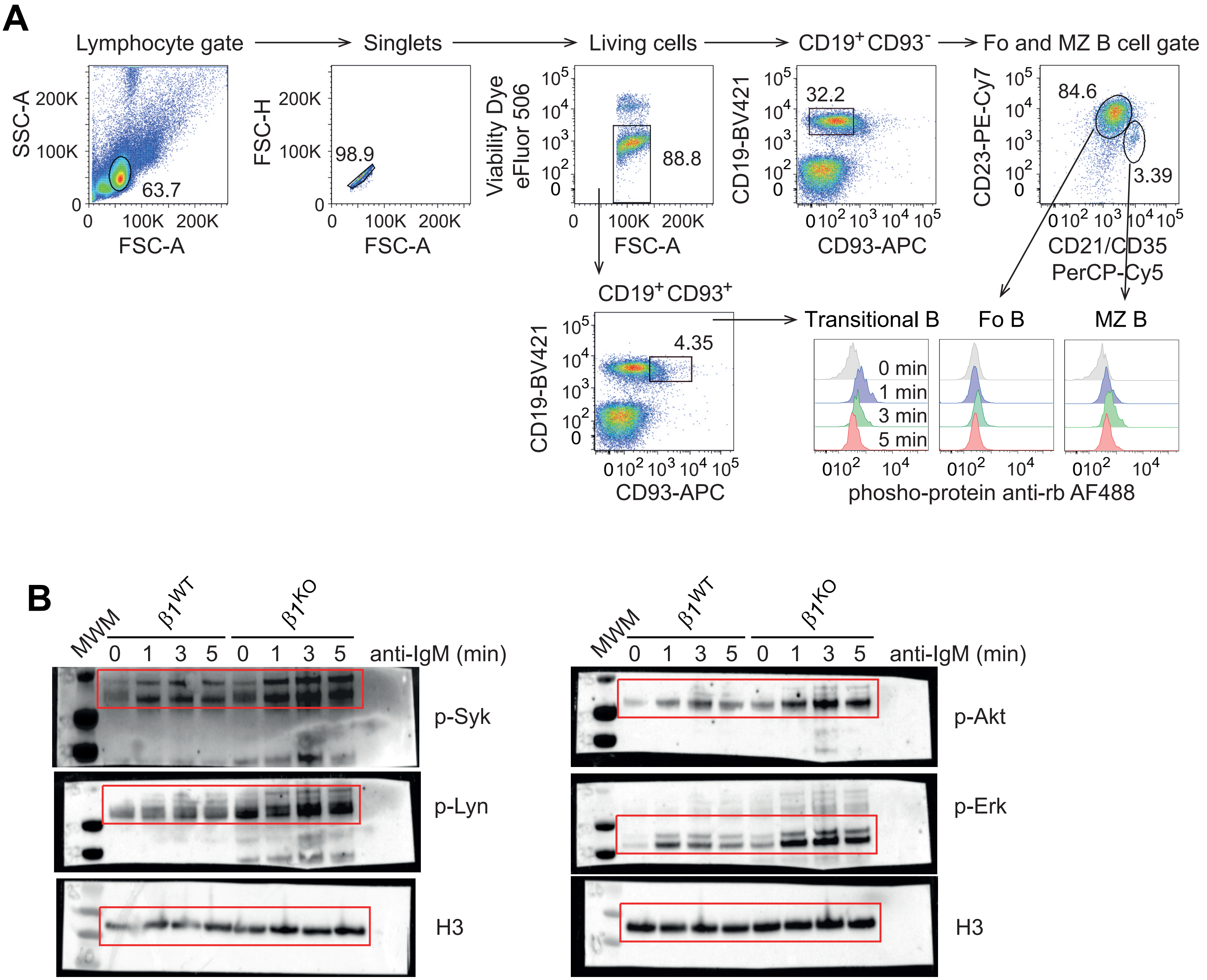

(A) General and representative flow cytometry gating strategy for the analysis of phospho-proteins in Transitional, Fo and MZ B cells. (B) Raw data for Western Blot. Prior to antibody incubation, blots were cut between 37-75 kDa and 10-25 kDa marker band. Precision Plus Protein Dual Color Standards (Biorad: 161-0374) was used for protein Standard (MWM: Molecular Weight Marker). Red rectangles correspond to samples and cropped blot included in the manuscript.
Representative of n=3 independent experiments.
