# Supplementary material for: Integrin β1 regulates marginal zone B cell differentiation and PI3K signaling
Source: J Exp Med. 2022 Nov 9;220(1):e20220342. doi: 10.1084/jem.20220342 (PMC9814157; doi:10.1084/jem.20220342)
Supplement: SourceData F8 — contains original blots for Fig. 8. [file JEM_20220342_SourceDataF8.pdf]

Andreani_Source Data_Fig 8

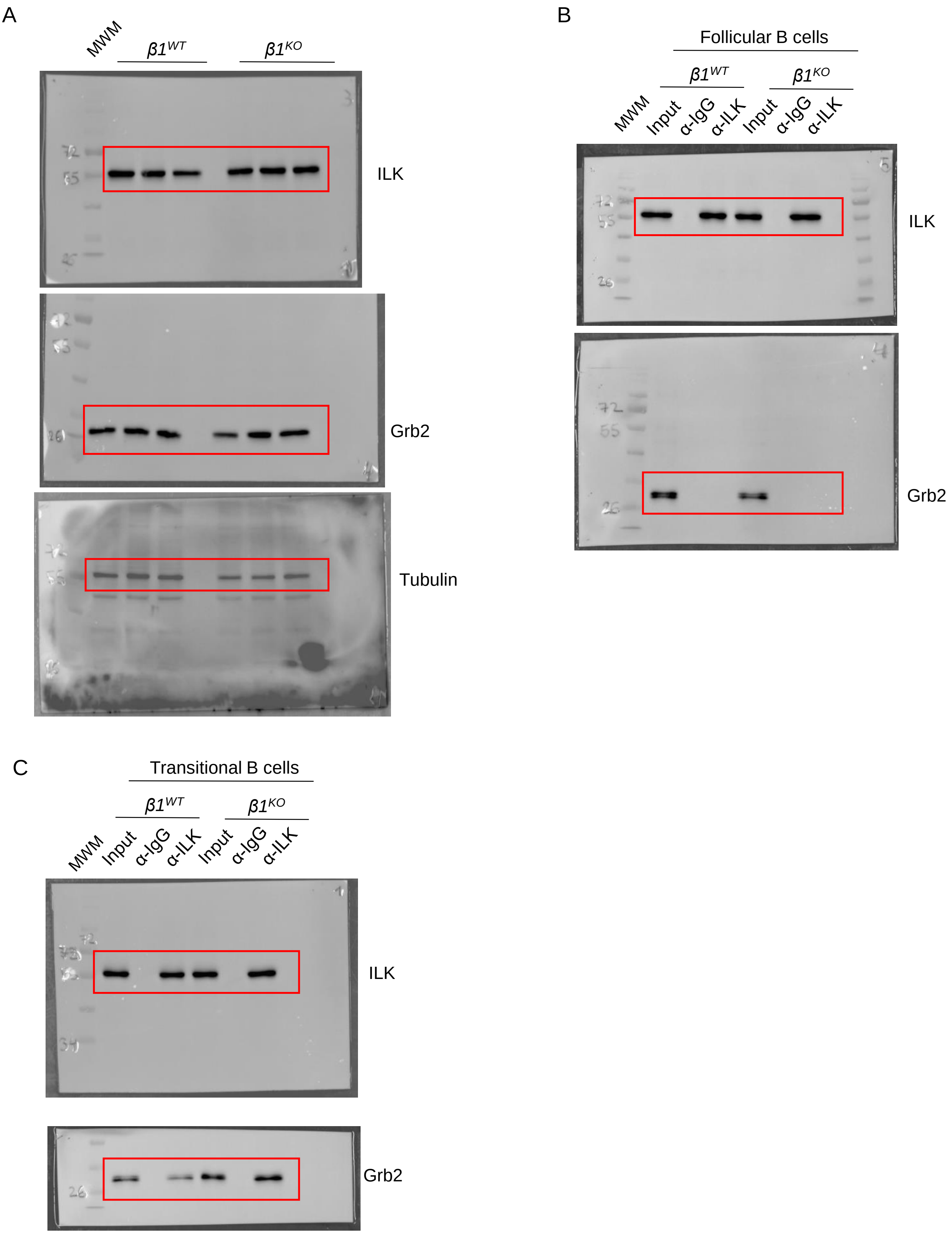

Raw data for Immunoblot and Co-immunoprecipitation. (A) Immunoblot analysis to detect ILK and Grb2 in transitional B cells. The Grb2 immunoblot was re-probed to detect Tubulin as a loading control. Each lane represents a different mouse. (B-C) Co-immunoprecipitation of Grb2 with anti-ILK in lysates from follicular B cells (B) and transitional B cells (C). Color-Prestained Protein Standard, Broad Range (Biolabs: P7719S) was used as protein standard (MWM: Molecular Weight Marker). Red rectangles correspond to the cropped area included in Figure 8. Blots are representative of three independent experiments.
