# Supplementary material for: Integrin β1 regulates marginal zone B cell differentiation and PI3K signaling
Source: J Exp Med. 2022 Nov 9;220(1):e20220342. doi: 10.1084/jem.20220342 (PMC9814157; doi:10.1084/jem.20220342)
Supplement: SourceData FS4 — contains original blots for Fig. S4. [file JEM_20220342_SourceDataFS4.pdf]

Andreani_Source Data_Fig S4

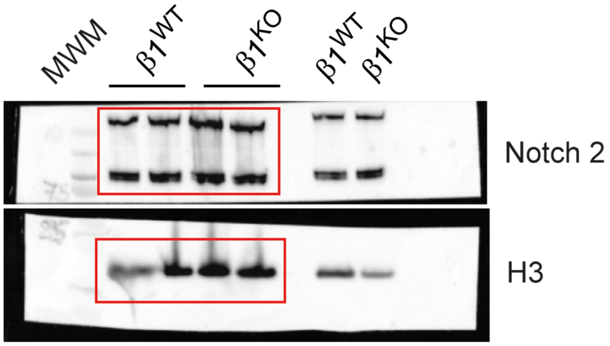

Raw data for immunoblot analysis
Prior to antibody incubations, blots were cut at the 250kDa, 75kDa, 25kDA and 10 kDa molecular weight markers (MWM). The blot section between 250 kDa and 75 kDa was incubated with anti-Notch2 antibody and the section between 25 kDa and 10 kDa was incubated with anti-H3 antibody. Precision Plus Protein Dual Color Standards (Biorad: 161-0374) was used as protein standard. Red boxes correspond to the cropped area included in Supplemental Figure S4. Blot is representative for three experiments.
