# Supplementary material for: Integrin β1 regulates marginal zone B cell differentiation and PI3K signaling
Source: J Exp Med. 2022 Nov 9;220(1):e20220342. doi: 10.1084/jem.20220342 (PMC9814157; doi:10.1084/jem.20220342)
Supplement: SourceData FS5 — contains original blots for Fig. S5. [file JEM_20220342_SourceDataFS5.pdf]

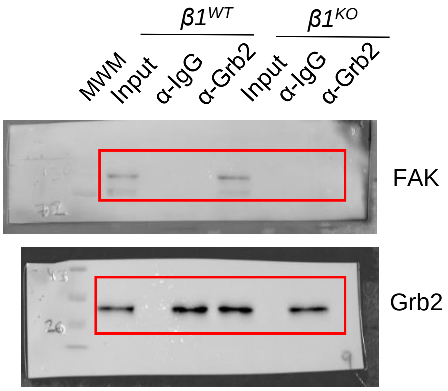

Raw data of co-immunoprecipitation of Grb2 and FAK. FAK was co-immoprecipitated with anti-Grb2 antibody in lysates of transitional B cells. Immunoblots were probed with anti-FAK or anti-Grb2 antibodies. Color Prestained Protein Standard, Broad Range (Biolabs: P7719S) was used for protein Standard (MWM: Molecular Weight Marker). Red rectangles correspond to the cropped areas included in Supplemental Figure S5. Blots are representative of three independent experiments.
